# Inhibition of KLF5 promotes ferroptosis via the ZEB1/HMOX1 axis to enhance sensitivity to oxaliplatin in cancer cells

**DOI:** 10.1038/s41419-025-07330-8

**Published:** 2025-01-18

**Authors:** Zheng Zhang, Huaxiang Xu, Junyi He, Qiangsheng Hu, Yuxin Liu, Zijin Xu, Wenhui Lou, Wenchuan Wu, Lei Zhang, Ning Pu, Chenye Shi, Yaolin Xu, Wenquan Wang, Liang Liu

**Affiliations:** 1 Department of Pancreatic Surgery, Zhongshan Hospital, Fudan University, Shanghai, China; 2 Cancer Center, Zhongshan Hospital, Fudan University, Shanghai, China; 3 Department of General Surgery, Zhongshan Hospital, Fudan University, Shanghai, China; 4 Department of Thoracic Surgery, Shanghai Pulmonary Hospital, Tongji University School of Medicine, Shanghai, China; 5 Institute of liver diseases, Shanxi Medical University, Taiyuan, Shanxi China; 6 Department of General Surgery, Qingpu Branch of Zhongshan Hospital Affiliated to Fudan University, Shanghai, China

**Keywords:** Chemotherapy, Cell death

## Abstract

As a novel form of nonapoptotic cell death, ferroptosis is developing into a promising therapeutic target of dedifferentiating and therapy-refractory cancers. However, its application in pancreatic cancer is still unknown. In the preliminary research, we found that F-box and WD repeat domain-containing 7 (FBW7) inhibited the migration and proliferation of pancreatic cancer cells through its substrate c-Myc. We further found that another key substrate of FBW7, KLF5, could inhibit ferroptosis. Inhibiting KLF5 significantly enhances the cytotoxicity of oxaliplatin rather than other chemotherapy drugs. Mechanistically, we found that KLF5 inhibited the expression of heme oxygenase 1 (HMOX1) via repressing zinc finger E-box-binding homeobox 1 (ZEB1). Inhibition of KLF5 facilitated the cytotoxic effect of oxaliplatin via promoting ferroptosis. Oxaliplatin combined with KLF5 inhibitor significantly potentiated cell death in vitro and inhibited tumor growth in vivo compared with either treatment alone. These results reveal a critical role of KLF5 in sensitized chemotherapy of pancreatic cancer, and suggest that ferroptosis combined with platinum-based chemotherapy rather than gemcitabine-based chemotherapy is expected to bring better therapeutic effects.

## Introduction

Pancreatic ductal adenocarcinoma (PDAC) is projected to be the second leading cause of cancer-related mortality by 2040 [1, 2]. Surgery and chemotherapy are the primary treatment strategies for PDAC. However, radical surgery can only be performed on 15–20% of patients, owing to the high incidence of distant metastasis at diagnosis. Less than 50% of patients are sensitive to chemotherapy [3, 4]. Moreover, chemotherapy has a limited effect on the survival of advanced pancreatic cancer and chemotherapy resistance is an important cause. Therefore, there is an urgency to develop novel strategies that sensitize cancer cells to chemotherapy.

Our prior study found that FBW7 regulated pancreatic cancer cell proliferation and metastasis through the substrate c-Myc [5]. Accumulating evidence has demonstrated that FBW7 acts as a crucial tumor suppressor by mediating the degradation of multiple substrates, including KLF5 [6, 7]. KLF5 belongs to the Krüppel-like factor family, which are evolutionarily conserved zinc finger-containing transcription factors with multiple regulatory functions in diverse human cancers [8]. KLFs regulate cancer cell apoptosis and proliferation, metastasis, cancer stem cells, and tumor microenvironment [9]. Emerging evidence has also identified KLF5 as an oncoprotein in various human malignancies, such as esophageal squamous carcinoma [10], colorectal cancer [11], prostate cancer [12], esophageal adenocarcinoma [13], and lung cancer [14]. KLF5 plays a crucial role in the carcinogenesis and development of PDAC. David et al. reported that KLF5 assisted Sox4 in tumorigenesis and repressed Sox4-induced apoptosis in PDAC [15]. Ablation of KLF5 in pancreatic cancer cells can alter the tumor immune microenvironment and sensitize tumors cells to combination immunotherapy [16]. KLF5 is also involved in proliferation, glycolysis, and immune cell infiltration of PDAC [17, 18]. However, the role of KLF5 in PDAC ferroptosis remains unclear.

Ferroptosis is a novel form of cell death characterized by iron-dependent phospholipid peroxidation. It has been demonstrated in various human diseases including cancer, neurodegenerative disorders, ischemia–reperfusion injury, and tissue damage during cold exposure [19–22]. In particular, ferroptosis activators have significant cytotoxic effects for tumor cells in vitro and immunodeficient mouse models [23, 24]. Induction of ferroptosis in the context of cancers has emerged as a promising strategy that suggests synergistic effects with cancer immunotherapy and even significantly kills metastatic and therapy-resistant cancers [25–29]. The relationship between ferroptosis and Ras mutation has a long history. Nearly 30% of cancers are accompanied by a mutation in the RAS family of small GTPases (HRAS, NRAS, and KRAS) [30]. Dolma et al. and Yang et al. screened small molecules that were selectively fatal to RAS mutant cell lines. They found two compounds, named Erastin and RAS-selective lethal 3 (RSL3), which could trigger an iron-dependent, nonapoptotic cell death [31, 32]. KRAS mutation occurs in ~95% of pancreatic tumors [33]. Recently, accumulating evidence has suggested a vital role of ferroptosis in PDAC cells [34–36]. Michael et al. also found that deletion of SLC7A11 mediated tumor-selective ferroptosis and suppressed PDAC growth [37]. Therefore, ferroptosis may be a promising approach to combat pancreatic cancer.

In this study, we investigated the correlation between upregulated KLF5 expression in PDAC tissues and poor prognosis. We also explored the impact of KLF5 on ferroptosis in PDAC. Our results indicated that KLF5 inhibited ferroptosis by repressing the ZEB1 / HMOX1 axis. Eventually, inhibition of KLF5 potentiated the cytotoxic effect of oxaliplatin in pancreatic cancer. Ferroptosis is evolving as a promising approach to killing difficult-to-treat tumors. Our findings might provide new strategies for the systemic treatment of pancreatic cancer.

## Materials and methods

### Cell culture

Six pancreatic cancer cell lines (PANC-1, MiaPaCa-2, CFPAC-1, Capan-1, BxPC3, and AsPC-1) and the human pancreatic duct epithelial cell line (HPNE) were obtained from the American Type Culture Collection. All cells were confirmed by DNA fingerprinting and passaged in our laboratory for <6 months after their receipt. PANC-1 and HPNE cells were maintained in Dulbecco’s modified Eagle’s medium (DMEM) containing 10% fetal bovine serum (FBS). CFPAC-1 cells were cultured in Iscove’s modified Dulbecco’s medium (IMDM) supplemented with 10% FBS. BxPC3 and AsPC-1 cells were cultured in RPMI 1640 medium supplemented with 10% FBS. Capan-1 cells were cultured in IMDM containing 20% FBS. MiaPaCa-2 cells were maintained in DMEM containing 10% FBS and 2.5% horse serum. All of the media was supplemented with 100 U/mL penicillin and 0.1 mg/mL streptomycin.

### Small compounds

Ferroptosis inhibitor, Fer-1, and ferroptosis inducers, Erastin and RSL3, were obtained from Selleckchem. ML264, hemin, gemcitabine, paclitaxel, 5-fluorouracil (5-FU), irinotecan, and oxaliplatin were purchased from MedChemExpress.

### Cell viability and cell death

Cells (4 × 10^3^) were maintained in 96-well plates and treated with corresponding small molecules. After incubation for 48–72 h, cell viability was evaluated using calcein-AM (Beyotime) and detected by synergy H4 (BioTek). Cell death was evaluated using propidium iodide (Beyotime) and analyzed by fluorescence-activated cell sorting (Beckman Coulter).

### Plasmids

The coding sequences of human KLF5 were cloned into the lentiviral vector pCDH-CMV-MCS-EF1-puro (SBI) to generate KLF5 expression plasmids. The pLKO.1 TRC cloning vector (Addgene) was utilized to establish shRNA plasmids against KLF5, HMOX1, and ZEB1, as previously described [38] Targets (21 bp) against KLF5 were CCTATAATTCCAGAGCATAAA and CCCTGAGTTCACCAGTATATT. Targets (21 bp) against HMOX1 were ACAGTTGCTGTAGGGCTTTAT and GCTGAGTTCATGAGGAACTTT. Targets (21 bp) against ZEB1 were CCTCTCTGAAAGAACACATTA and CGGCGCAATAACGTTACAAAT.

### Western blotting (WB)

Total cell protein lysates were separated by SDS-PAGE after extraction and blotted onto polyvinylidene fluoride membranes (Bio-Rad). After blocking using 5% BSA, the membranes were incubated with corresponding antibodies: KLF5, ZEB1, HMOX1, β-actin (Proteintech), GJA1, HNF4A, and HDAC1 (Abclonal).

### RNA isolation and quantitative real-time PCR (qRT-PCR)

The TRIzol reagent (Invitrogen) was used to extract total RNA. cDNA was prepared by reverse transcription utilizing the TaKaRa PrimeScript RT Reagent Kit. Quantitative real-time PCR was performed using an ABI 7900HT Real-Time PCR system (Applied Biosystems). Primers are shown in Supplementary Table S1.

### Clinical samples and immunohistochemical (IHC) staining

The clinical tissue samples were obtained from patients diagnosed with pancreatic cancer at Zhongshan Hospital, Fudan University (ZSHFU). The patients’ consent and approval from the Institutional Research Ethics Committee were obtained (Batch number:1310128-2). IHC staining was conducted as described previously [39]. A scoring scale was utilized to analyze the staining intensity (0, negative; 1, low; 2, moderate; 3, strong) and the percentage of stained cells (0, <10%; 1, 10–25%; 2, 25–50%; 3, 50–75%; 4, >75%). The product of two scores (frequency × intensity) was considered total scores. An immunohistochemical score >6 was defined as a high expression, whereas a score ≤6 was considered a low expression level. Paraffin sections underwent the standardized process of gradual deparaffinization, antigen retrieval, endogenous peroxidase elimination, blocking, antibody incubation, confocal imaging, and scanning by Servicebio, Wuhan, China. Anti-KLF5 (1:400), anti-ZEB1 (1:200), and anti-HMOX1 (1:200) (all Proteintech) were used to evaluate protein expression.

### Chromatin immunoprecipitation assay (ChIP)

The EZ-ChIP Kit (Millipore) was utilized to conduct ChIP assays following the manufacturer’s protocol. Primers to test HMOX1 promoter occupancy are displayed in Supplementary Table S2.

### Promoter activity detection by a dual-luciferase assay

The HMOX1 promoter region, spanning from −2000 to +100 of the transcription start site or relevant mutant sequence, was amplified from genomic DNA and cloned into the pGL3-Basic vector. A dual-luciferase system (Promega) was utilized to detect firefly and Renilla luciferase activities following the manufacturer’s protocol.

### Lipid peroxidation assay

BODIPYTM 581/591 C11 (D3861; Thermo Fisher Scientific) was utilized to detect lipid peroxidation. After oxidation, the fluorescence emission peak of BODIPY-C11 shifted from 590 to 510 nm, which was proportional to the production of lipid reactive oxygen species (ROS). After pretreatment with test agents for the indicated times, cells were dissociated, resuspended, washed, and stained with 2 μmol/L BODIPYTM 581/591 C11 for 30 min. These cells were measured and analyzed utilizing a flow cytometer, and data were fetched from the FL1 channel. For confocal imaging, cells were seeded on round coverslips. Before detection, cells were stained with 2 μmol/L BODIPYTM 581/591 C11 for 30 min. These cells were washed and fixed with 4% paraformaldehyde (Thermo Fisher Scientific). The images were obtained using confocal microscopy.

### Malondialdehyde (MDA) assay

PDAC cells were seeded in six-well culture plates. The concentration of protein was detected using the BCA protein assay kit (Beyotime) after cell homogenization. MDA was detected using the lipid peroxidation MDA assay kit (Beyotime). The ratio of MDA to protein concentration was analyzed based on the concentration of MDA.

Tumors were extracted after all of the mice were killed. The tissue samples were homogenized and sonicated in precooled RIPA buffer on ice. After centrifugation at 12,000×*g* for 15 min at 4 °C, the supernatant was obtained and submitted to the MDA assay.

### Transmission electron microscopy (TEM)

CFPAC-1 and Capan-1 cells were seeded in 10-cm cell culture dishes (Corning) and prepared with Fer-1 (2 μmol/L) or DMSO for 24 h. The cells were collected and fixed using 2.5% glutaraldehyde. TEM was performed by Servicebio.

### Sphere-formation assay

The cells were diluted to a density of 1000 cells/ml with the serum-free media (SFM). The SFM is DMEM-F12 (1:1) added with B27 (1:50) (Invitrogen), 20 ng/ml epidermal growth factor (R&D Systems), 2 mmol/l l-glutamine (Invitrogen) and 10 ng/ml basic fibroblast growth factor (R&D Systems). The cells (100 cells/well) were seeded into a 96-well low attachment plate. Each well was replaced with fresh SFM at day 7. The spheres larger than 50 μm were counted under a light microscope at day 15.

### Labile iron pool staining

The cells (10^4^ cells/well) were seeded into a 24-well plate and cultivated for 24 h. Processed adherent cells were stained with Ferroorange. Labile iron pool could be stained by Ferroorange (Servicebio). In brief, the cells were incubated in the complete medium added with ferroorange (1 μM) for 30 min in a cell culture incubator. Then, the cells were observed and photographed using an inverted fluorescence microscope (Leica).

### Total iron concentration assay

The harvested cells were homogenized and lysed, then centrifuged to collect the supernatant. The iron ion standard was diluted to certain concentrations according to the manufacturer’s instructions (Servicebio). The probe was then mixed with the sample at 37 °C for 40 min. Then the mixture was centrifuged at 10,000× *g* for 5 min and the absorbance value of the collected supernatant was detected at 593 nm. A standard curve was established using absorbance values of different concentration standards. The total iron concentration of samples could be calculated according to the standard curve.

### Predicting transcription factor binding sites

The promoter sequences of the target genes were found first at NCBI. Then the sequences were entered into the Jaspar website and the transcription factor was selected as KLF5 or ZEB1. Using a relative scoring thresholdå 80% as cut-off criteria and the binding sites with the top five scores were further confirmed experimentally.

### Animal studies

Five-week-old male nude mice were purchased from Shanghai SLAC Laboratory. About 5 × 10^6^ cells were subcutaneously inoculated into the right flank of the mice until the tumor volume reached ~100 mm^3^. The mice were randomly divided into four subgroups of five each: PBS, oxaliplatin, ML264, and combination of oxaliplatin and ML264. Intraperitoneal (i.p.) injections of oxaliplatin (6 mg/kg) were administered weekly. ML264 (20 mg/kg, i.p.) was conducted once daily. Next, tumor size was measured every 3 days and the tumor volume was calculated as length × width^2^ × 0.5. At 5 weeks post-implantation, the tumor samples were surgically dissected and treated for histological assessment. The patient-derived xenograft (PDX) model was established following a protocol: tumor cells derived from PDX models previously developed by our research group were revived and then injected subcutaneously into 5-week-old BALB/c nude mice. Once palpable tumors formed, the same procedures—including tumor measurement and drug administration—were applied to treat the mice with PDX. The protocol was approved by the Committee on the Ethics of Animal Experiments of Fudan University and conformed to the Guide for the Care and Use of Laboratory Animals published by the National Institutes of Health.

### Cell proliferation and colony formation assays

To assess cell proliferation, cell viability was measured using the CCK-8 assay (Dojindo Molecular Technologies). At each experimental time point, 10 µL of CCK-8 solution was added to each well of a 96-well plate containing seeded cells, followed by a 2-h incubation. Absorbance at 450 nm was then measured using a multimode microplate reader. For IC_50_ assessment, cells were seeded in 96-well plates and treated with the specified inhibitors. Viable cell counts were obtained using the CCK-8 kit, with absorbance recorded at 450 nm on a spectrophotometer. Cytotoxicity (%) = [1 − (absorbance of the experimental well − absorbance of the blank)/(absorbance of the untreated control well − absorbance of the blank)] × 100. The IC_50_ was determined from the concentration–response curve. For the colony formation assay, cells were plated in six-well plates and cultured for 10 days. Afterward, cells were fixed with 4% paraformaldehyde and stained with 1% crystal violet. Colonies were subsequently counted.

### Statistical analysis

The experiments were repeated at least three times. All data were processed by SPSS version 22.0 (IBM) or GraphPad Prism 8. The differences between any two groups were evaluated using two-tailed unpaired Student *t* tests. The survival curve was compared by the log-rank test and plotted by the Kaplan–Meier method. The correlation of KLF5 with various molecules was analyzed using Spearman Correlation Analysis based on mRNA expression level and the results were shown as a lollipop chart. The | logFc | > 1.0 and adj *P* < 0.05 were used as cut-off criteria, and the differentially expressed genes in transcriptome sequencing were selected for KEGG enrichment analysis using the clusterProfiler package in R language. The significance of enrichment was analyzed using hypergeometric distribution, with Benjamini–Hochberg correction and *P*.adjust <0.05 as cut-off criteria. The value of the Z-score was mapped by the lengths of the strips. *P* value was mapped by the color of the strips. Differences were considered statistically significant at *P < 0.05 and **P < 0.01, ***, *P* < 0.001, ****P < 0.0001, and ns meant there was no significant difference.

## Results

### KLF5 expression negatively correlates with pancreatic cancer prognosis

**Fig. 1:**
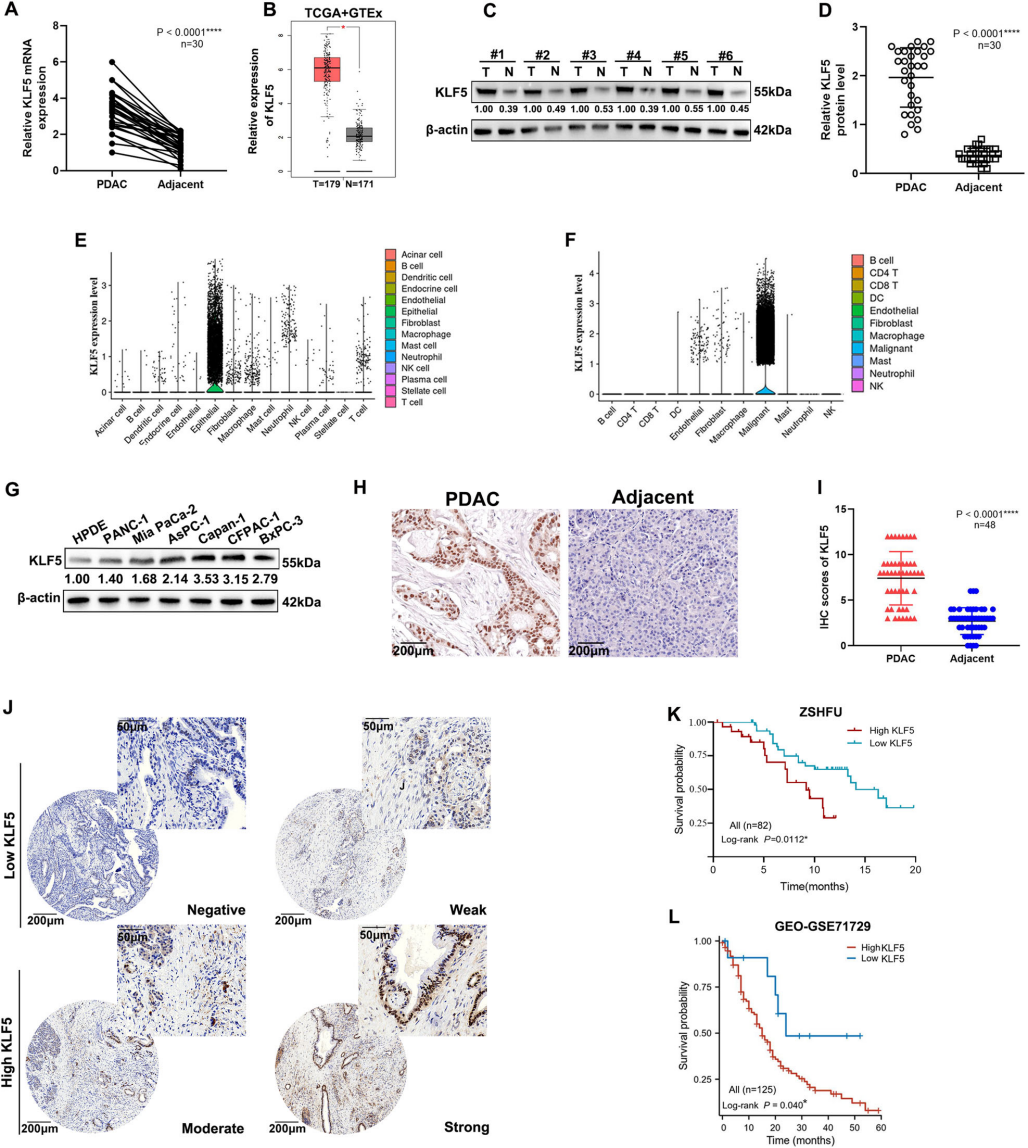
KLF5 expression is increased in PDAC tissues. **A** KLF5 mRNA expression levels in PDAC and para-cancerous tissue (*n* = 30, ****P < 0.0001). **B** KLF5 mRNA expression levels in PDAC from TCGA-PAAD and para-cancerous tissue matching TCGA normal and GTEx data (*P < 0.05). **C** Representative western blots in PDAC and para-cancerous tissue. **D** Data showing high expression of KLF5 in tumor tissues (*n* = 30, ****P < 0.001). **E**, **F** KLF5 expression across various clusters in PDAC from two single-cell RNA-sequencing datasets (GEO: GSE155698, GSE202051). **G** Western blots of KLF5 expression in HPDE and PDAC cell lines. **H** Representative images of KLF5 in IHC staining in PDAC and para-cancerous tissues (scale bar, 200 μm). **I** KLF5 expression in PDAC and para-cancerous tissues, as evaluated by IHC score (*n* = 48, ****P < 0.0001). **J** Representative images of IHC staining for KLF5 in TMAs (scale bar, 200 µm, 50 µm). **K** OS of PDAC patients was analyzed using Kaplan–Meier analysis according to KLF5 expression in our center (ZSHFU) (*n* = 82, *P = 0.0112). **L** OS of PDAC patients was evaluated using Kaplan–Meier analysis according to KLF5 expression (*n* = 125, *P = 0.04) from the PDAC dataset (GEO: GSE71729).

Among all 17 members of the KLF family, only expression of KLF5 and KLF15 was significantly correlated with overall survival (OS) and disease-free survival (DFS) simultaneously according to the TCGA database (Fig. S1A). Survival analysis showed that OS and DFS were significantly shorter in pancreatic cancer patients with high KLF5 expression. P value is significantly less than 0.05 (0.01; 0.023). Correspondingly, survival analysis of patients expressing KLF15 showed that the *P* values for OS and DFS were only slightly lower than 0.05 (0.042, 0.041). The intergroup differences in survival analysis were more significant for patients expressing KLF5 (Fig. S1B, C). We explored the expression of the KLF5 mRNA in 30 paired samples of PDAC and para-carcinoma tissue from patients. KLF5 expression was increased in PDAC tissues (Fig. 1A). Correspondingly, TCGA and GTEx data also indicated that KLF5 was highly expressed in pancreatic cancer (Fig. 1B). These findings were verified at the protein level by WB to investigate the level of KLF5 in 30 paired samples of pancreatic cancer and adjacent normal tissues (Fig. 1C, D). To measure the expression patterns among the overall cell populations in PDAC, we reanalyzed two published single-cell RNA-sequencing dataset in PDAC patients [40, 41], and found that KLF5 was expressed at its highest level in cancer cells (Fig. 1E, F). We analyzed the levels of KLF5 protein in HPDE cells and diverse PDAC cell lines. KLF5 expression was upregulated in pancreatic cell lines compared with the HPDE cells (Fig. 1G). Subsequent IHC results confirmed that KLF5 was expressed at higher levels in PDAC tissues than in para-carcinoma tissues (Fig. 1H, I). We validated the IHC results in tissue microarrays (TMAs) containing 82 pairs of PDAC patient samples (Fig. 1J). The log-rank test suggested a significant correlation between high KLF5 expression and poor prognosis of PDAC patients (*P* = 0.0112) (Fig. 1K) in our center (ZSHFU). The relationship between KLF5 expression and the prognosis of PDAC was validated using the GEO dataset (GSE71729) (*P* = 0.04) (Fig. 1L). We further examined the expression and prognostic significance of KLF5 in the top ten cancers with the highest mortality rates, in addition to pancreatic cancer. Our analysis revealed that KLF5 expression was significantly elevated in tumor tissues compared to adjacent normal tissues in cases of colon cancer, ovarian cancer, and cervical cancer (Fig. S1D). In addition, increased KLF5 expression was significantly associated with reduced OS in lung cancer (Fig. S1E).

### Inhibition of KLF5 potentiates ferroptosis in PDAC cells

**Fig. 2:**
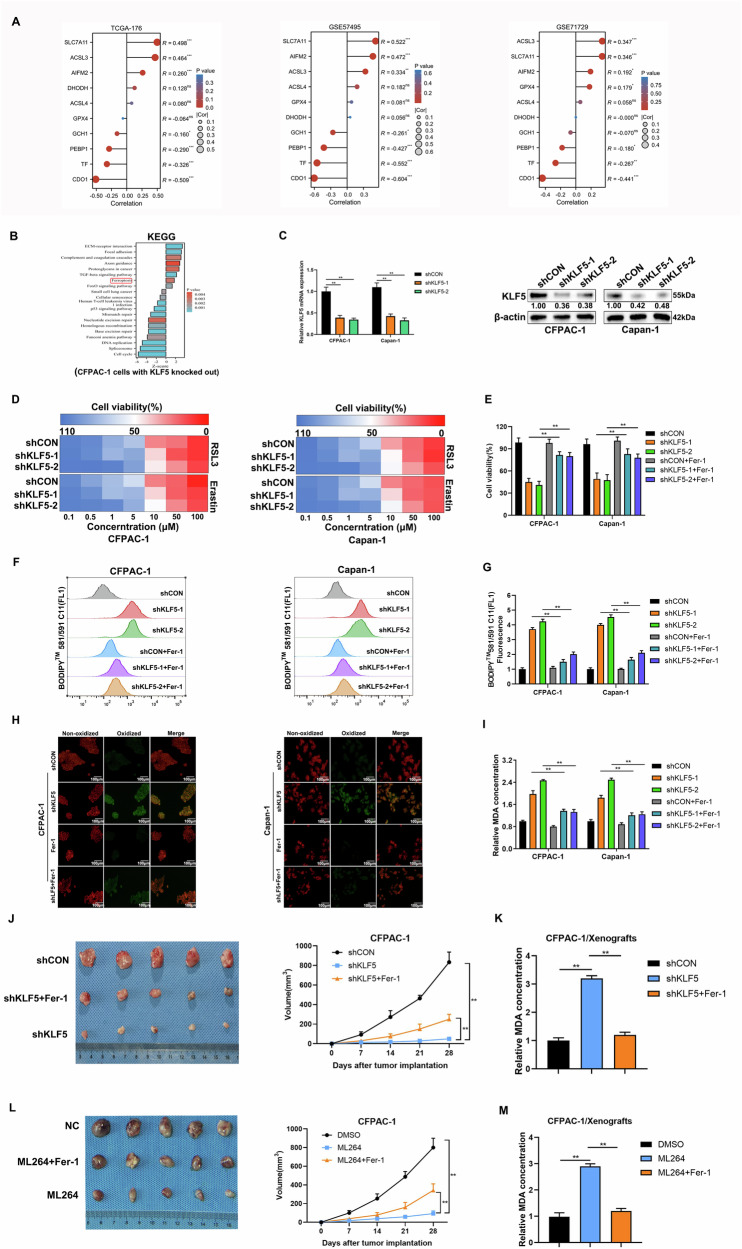
Inhibition of KLF5 promotes ferroptosis in pancreatic cancer cells. **A** The lollipop diagram depicted the correlation between KLF5 and ten key genes related to ferroptosis according to mRNA expression using Spearman analysis. The patients with pancreatic cancer come from three datasets: TCGA-PAAD, GEO (GSE57495), and GEO (GSE71729). **B** KEGG analysis of KLF5-targeted genes. **C** qRT-PCR evaluated knockdown efficiency of shKLF5. WB evaluated the knockdown efficiency of shKLF5. **D** Heatmap depicted inhibition of KLF5 increased the sensitivity to ferroptosis inducers RSL3 and Erastin. **E** Short hairpin RNA was used to silence KLF5 in CFPAC-1 and Capan-1 cells. The cells were treated with Fer-1 (2 μmol/L) for 16 h. Cell viability was detected by calcein-AM. **F** BODIPY 581/591 C11 was used to detect lipid peroxidation in CFPAC-1 and Capan-1 cells. **G** Fluorescence was calculated. **H** Confocal imaging revealed the effect of inhibition of KLF5 on lipid peroxidation. **I** MDA level was analyzed in KLF5-silenced CFPAC-1 and Capan-1 cells in the presence or absence of 2 μmol/L Fer-1. **J** Evaluation of shKLF5-treated tumors with or without Fer-1 (30 mg/kg) treatment. **K** MDA level in tumors. **L** Evaluation of ML264-inhibited tumors with or without Fer-1 (30 mg/kg) treatment. **M** MDA levels in tumors.

To explore the correlation between KLF5 and ferroptosis, we evaluated the correlation between KLF5 and ten critical ferroptosis genes utilizing TCGA-PAAD, GEO dataset (GSE57495, GSE71729). The results revealed that KLF5 was positively correlated with ferroptosis suppressor genes and was negatively related with ferroptosis inducer genes (Fig. 2A). Moreover, we reanalyzed the transcriptome sequencing in which KLF5 was knocked out in pancreatic cancer cell line CFPAC-1 [42]. Pathway enrichment indicated that inhibition of KLF5 promoted ferroptosis (Fig. 2B). To validate this, we constructed stable KLF5-silencing cell lines using CFPAC-1 and Capan-1 cells. The knockdown efficiency was confirmed by qRT-PCR and WB (Fig. 2C). We detected the killing effect of ferroptosis activators (RSL3 and Erastin) in CFPAC-1 and Capan-1 stable cell lines. Inhibition of KLF5 increased the killing effect of ferroptosis activators in CFPAC-1 and Capan-1 cells (Fig. 2D). Ferrostatin-1 (Fer-1), a ferroptosis inhibitor, also rescued cell viability suppressed by downregulated KLF5 (Fig. 2E). As ferroptosis was characterized by lipid peroxidation, we utilized a BODIPY 581/591 C11 probe to further measure lipid peroxidation through flow cytometry and confocal microscopy. Fluorescence changed from red to green when lipid peroxidation emerged. Suppression of KLF5 induced an increase in lipid peroxidation, which could be reversed by Fer-1 (Fig. 2F, G). The results were also demonstrated using confocal imaging (Fig. 2H). Inhibition of KLF5 increased the level of malondialdehyde (MDA), the end product of lipid peroxidation, which was impaired by Fer-1 (Fig. 2I). Through the subcutaneous injection of CFPAC-1 cells into three groups of nude mice (shCON, shKLF5, and shKLF5+Fer), the xenograft animal models verified the pro-ferroptosis effects of inhibition of KLF5. Suppression of KLF5 decreased the size of tumors (Fig. 2J) and enhanced MDA levels compared with the shCON group (Fig. 2K) and these were reversed by Fer-1. The in vivo results were confirmed by KLF5 inhibitor ML264 (Fig. 2L, M). In addition, overexpression of KLF5 (Fig. S2A, B) decreased the cytotoxic effects of Erastin in PANC-1 and MiaPaCa-2 cells (Fig. S2C, D). Upregulation of KLF5 decreased Erastin-mediated MDA (Fig. S2E, F) and lipid peroxidation (Fig. S2G–I). To analyze the effect of KLF5 on cells more intuitively, we performed TEM of PANC-1 and MiaPaCa-2 cells. Erastin induced smaller mitochondria and increased membrane density, which were reversed by overexpression of KLF5 (Fig. S2J, K). These indicated that inhibition of KLF5 enhanced ferroptosis in pancreatic cancer cells.

### Inhibition of KLF5 enhances ferroptosis by upregulating HMOX1

**Fig. 3:**
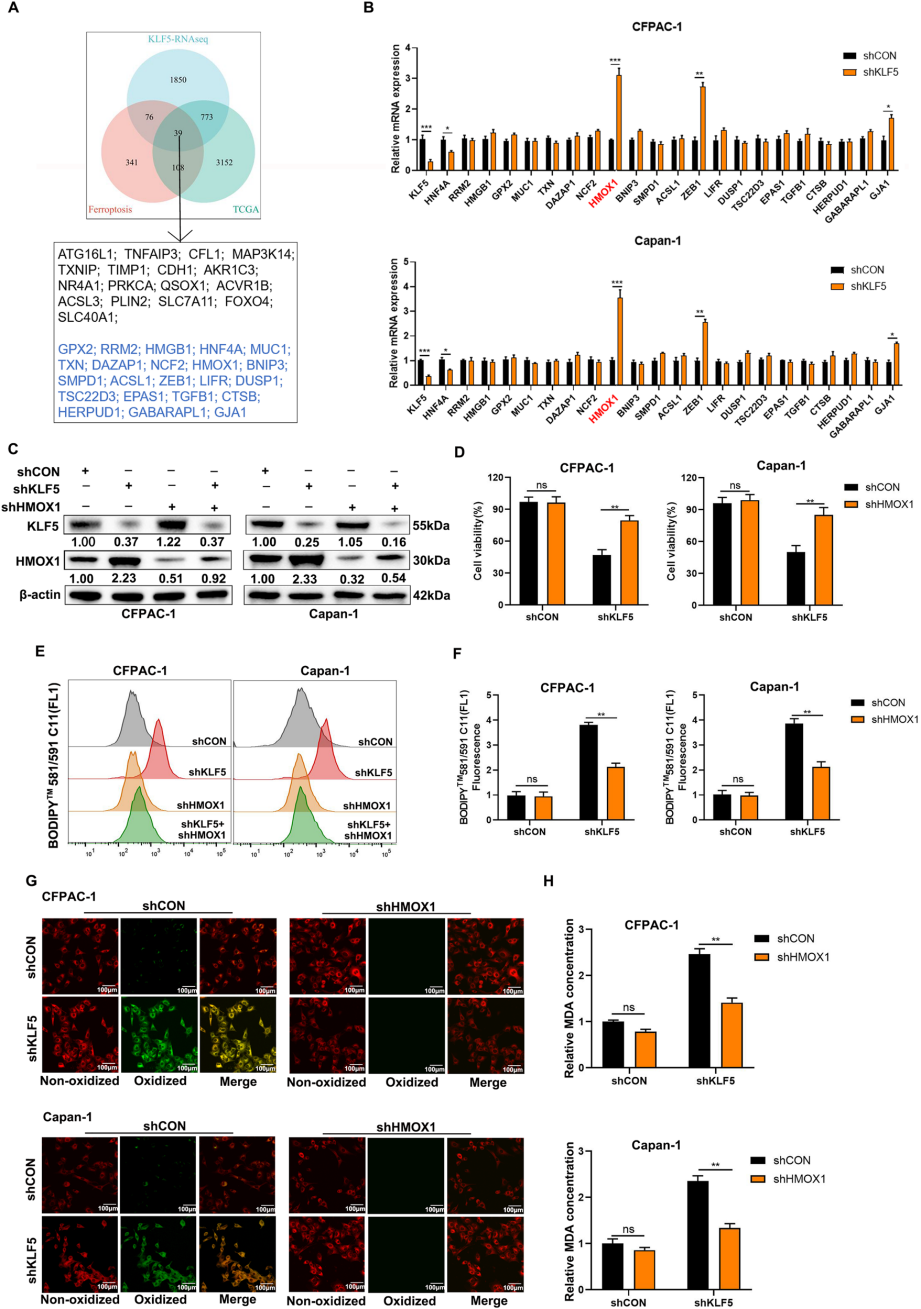
Silencing of KLF5 potentiates ferroptosis by upregulating HMOX1. **A** Venn diagram showing that the ferroptosis-related genes downstream of KLF5 intersected from the ferroptosis dataset and KLF5-mediated TCGA-PAAD cohort and transcriptome sequencing. **B** mRNA level in human PDAC cells treated by control plasmid or shKLF5. **C** WB showed the change in HMOX1 caused by individually or simultaneously suppressed KLF5 and HMOX1 in CFPAC-1 and Capan-1 cells. **D** KLF5 and HMOX1 in CFPAC-1 and Capan-1 cells were individually or simultaneously suppressed and cell viability was evaluated using calcein-AM. **E, F** BODIPY 581/591 C11 was used to detect lipid peroxidation in PDAC cells treated by shKLF5 or shHMOX1 or both. **G** Lipid peroxidation of PDAC cells treated by shKLF5 or shHMOX1 or both, detected using confocal microscopy. **H** MDA was detected in PDAC cells treated by shKLF5 or shHMOX1 or both.

We identified 39 candidate genes by intersecting the ferroptosis gene set from FerrDb [43] with the gene expression profiling array of the KLF5-mediated TCGA-PAAD cohort and transcriptome sequencing data (Fig. 3A). Phenotypic assays confirmed that inhibiting KLF5 promotes ferroptosis by upregulating ferroptosis-promoting genes or downregulating ferroptosis-inhibiting genes. Consequently, we initially excluded a total of 17 downregulated ferroptosis-promoting genes and upregulated ferroptosis-inhibiting genes following KLF5 inhibition, revealed by two transcriptome sequencing datasets (Fig. 3A). Among the 17 genes, only ATG16L1, CFL1, PLIN2, and NR4A1 have slight differences validated through qPCR analysis. (Fig. S3A). The remaining 22 candidate genes were further validated using qPCR. The qPCR results demonstrated that among these candidates, HMOX1 and ZEB1 showed the most significant expression differences in CFPAC-1 and Capan-1 cells (Fig. 3B), which was further confirmed by WB analysis (Fig. S3B). HMOX1 catalyzed the degradation of heme, which releases free iron and causes iron overload and ferroptosis [44], while ZEB1 functioned as a transcription factor (TF). Thus, HMOX1 was finally identified as the direct effector responsible for KLF5-mediated ferroptosis. To validate suppression of KLF5 enhancement of ferroptosis via HMOX1, we constructed stable KLF5-silencing and HMOX1-silencing CFPAC-1 and Capan-1 cells (Fig. 3C). Cell viability was inhibited by downregulated KLF5, which was reversed by repression of HMOX1 (Fig. 3D). Inhibition of HMOX1 reversed the enhanced lipid peroxidation induced by KLF5 inhibition (Fig. 3E, F). The results were also demonstrated using confocal imaging (Fig. 3G). In addition, the inhibition of HMOX1 reversed the enhanced level of MDA induced by KLF5 inhibition (Fig. 3H). The above results indicated that inhibition of KLF5 mediated ferroptosis through upregulated HMOX1.

### KLF5 suppresses HMOX1 transcription via ZEB1

**Fig. 4:**
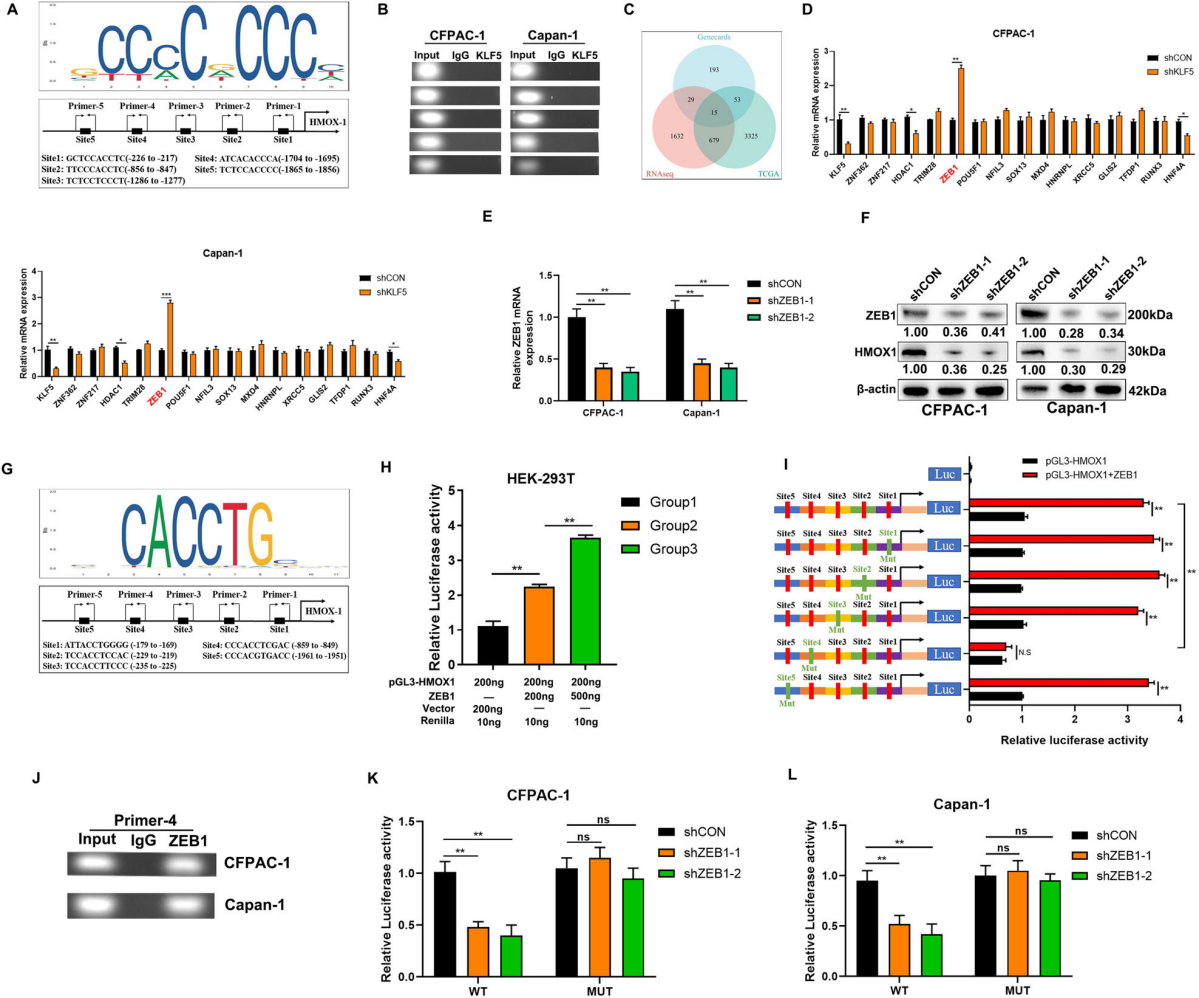
KLF5 suppresses HMOX1 transcription via ZEB1. **A** The binding sites (−2000 to +100) of KLF5 in the HMOX1 promoter region predicted by the JASPAR matrix model. **B** ChIP assay showed that KLF5 could not occupy the binding sites of the HMOX1 promoter region. **C** Genecards predicted all potential transcription factors upstream of HMOX1 and Venn diagram showing that the transcription factors downstream of KLF5 intersected from genecards and KLF5-mediated TCGA-PAAD cohort and transcriptome sequencing. **D** mRNA level in human PDAC cells treated by control plasmid or shKLF5. **E**, **F** HMOX1 changes were measured in ZEB1-silenced CFPAC-1 and Capan-1 cells. **G** The binding sites (−2000 to +100) of ZEB1 in the HMOX1 promoter region predicted by the JASPAR matrix model. **H** ZEB1 upregulated HMOX1 promoter activity in HEK-293T cells in a dose-dependent manner. **I** To evaluate the activation of the HMOX1 promoter regulated by ZEB1, the ZEB1 plasmid and promoter constructs were co-transfected into HEK-293T cells for 24 h. The bar chart shows the promoter activities of site mutagenesis on predicted binding sites. The luciferase activity is shown as the fold activated by ZEB1 compared with the controls. **J** ZEB1 occupied the binding sites of the HMOX1 promoter region in CFPAC-1 and Capan-1 cells, as measured by ChIP assay. **K**, **L** Dual-luciferase reporter system was used to confirm the effect of ZEB1 on HMOX1 promoter activity in PDAC cells treated by shZEB1 or control plasmid.

As KLF5 inhibited HMOX1 at the transcriptional level and KLF5 acted as a transcription factor, we first supposed that HMOX1 was the target gene of KLF5. The potentially upstream promoter region (−2000 to 100) of HMOX1 was evaluated using JASPAR [45]. The top five potential binding sites were selected for the next step of detection (Fig. 4A). ChIP results demonstrated that KLF5 was not bound to the HMOX1 promoter (Fig. 4B). Thus, HMOX1 may not be the direct target gene of KLF5, and KLF5 might mediate the transcription of a transcription factor, which binds to the HMOX1 promotor to enhance HMOX1 transcription. Subsequently, potential upstream TFs of HMOX1 were predicted utilizing Genecards (https://www.genecards.org/). We then intersected predicted TFs with differentially expressed genes from the KLF5-mediated TCGA-PAAD cohort and transcriptome sequencing, and identified 15 TFs (Fig. 4C). Among the 15 genes, ZEB1 exhibited the most pronounced difference in expression in CFPAC-1 and Capan-1 cells. (Fig. 4D). which be validated by WB (Fig. S3B). To verify that ZEB1 regulated HMOX1, we established stable ZEB1-silenced pancreatic cancer cells. qPCR and WB revealed that ZEB1 silencing inhibited HMOX1 expression (Fig. 4E, F). We predicted the top five potential binding sites using JASPAR (Fig. 4G), and found that ZEB1 enhanced HMOX1 promoter activity in a dose-dependent manner (Fig. 4H). Five HMOX1 promoter regions with or without distinct mutations were cloned into the pGL3 basic vector to conduct promoter analysis (Fig. 4I). The promoter activity of the wild-type (WT) HMOX1 construct was increased by ZEB1 overexpression; mutation (Mut) of the site4 completely reversed the stimulatory effect of ZEB1, whereas mutation of other sites had only a small effect on ZEB1-induced HMOX1 promoter activity (Fig. 4I). ChIP assay confirmed that ZEB1 was specifically bound to the HMOX1 promoter (Fig. 4J). ZEB1 silencing decreased the wild-type promoter activity of HMOX1 but not the mutant promoter in CFPAC-1 and capan-1 cells (Fig. 4K, L). Collectively, KLF5 inhibited HMOX1 transcription by decreasing the binding of ZEB1 to HMOX1 promoter.

### Ferroptosis mediated by inhibition of KLF5 depends on ZEB1

**Fig. 5:**
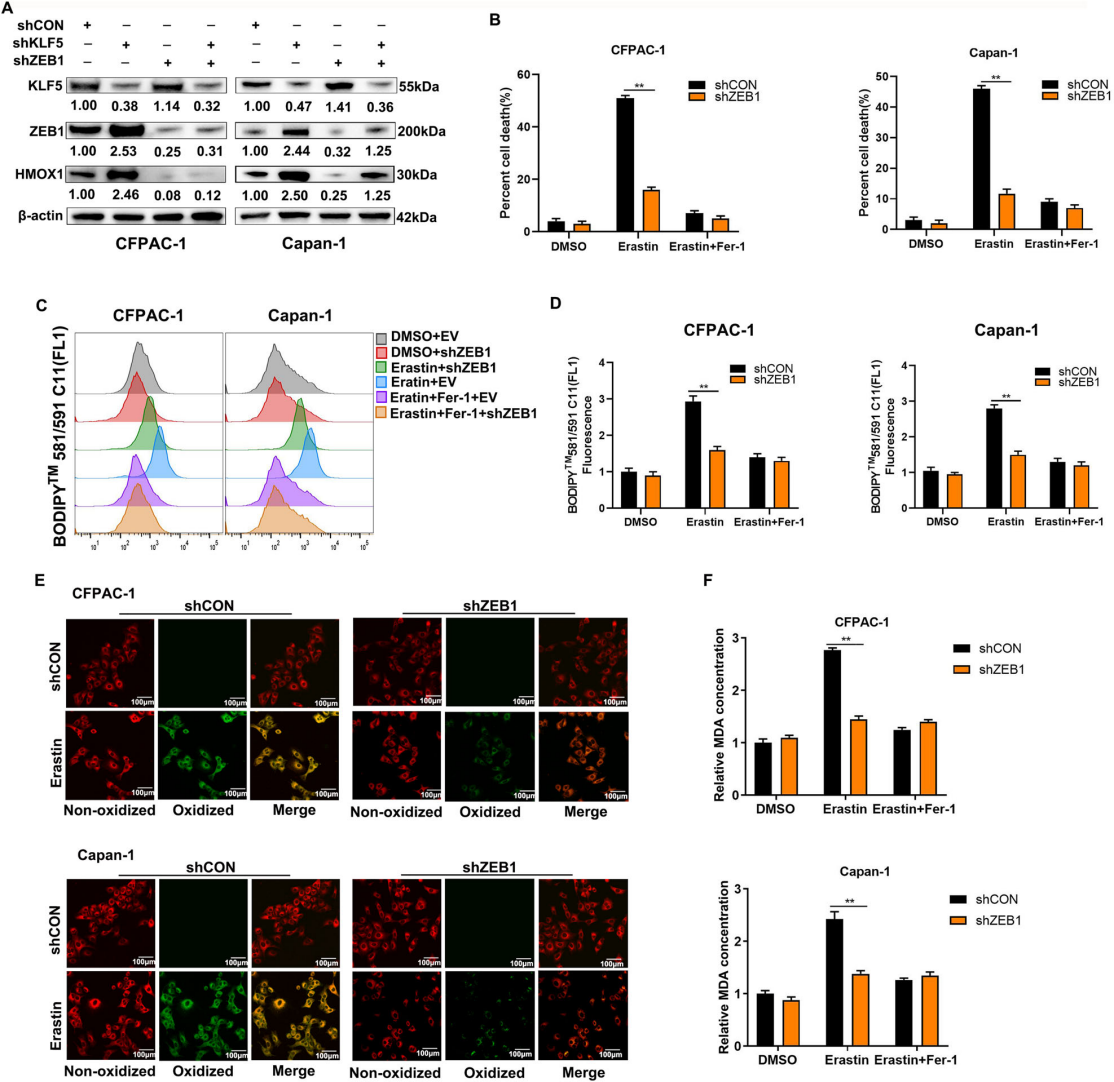
KLF5 relies on ZEB1 to regulate ferroptosis. **A** HMOX1 protein was detected in CFPAC-1 and Capan-1 cells treated by shKLF5 or shHMOX1 or both. **B** ZEB1-downregulated CFPAC-1 and Capan-1 cells were treated with 2.5 μmol/L Erastin and/ or 2 μmol/L Fer-1 for 16 h and cell death was analyzed by PI labeling. **C**, **D** CFPAC-1 and Capan-1 cells were subjected to the same treatment, and lipid peroxidation was detected using BODIPY 581/591 C11. **E** CFPAC-1 and Capan-1 cells were subjected to the same treatment and lipid peroxidation was analyzed using confocal microscopy. **F** CFPAC-1 and Capan-1 cells were subjected to the same treatment and the lipid peroxidation was analyzed using MDA.

We evaluated the role of ZEB1 in KLF5-regulated ferroptosis. We constructed stable KLF5-silencing and ZEB1-silencing CFPAC-1 and Capan-1 cells. WB confirmed that KLF5 regulated expression of HMOX1, which depended on ZEB1 (Fig. 5A). Downregulation of ZEB1 conferred resistance to Erastin in PDAC cells (Fig. 5B). The increased level of lipid peroxidation induced by Erastin was reversed by inhibition of ZEB1 using BODIPY-C11 (Fig. 5C, D). The results were also demonstrated using confocal imaging (Fig. 5E). Similarly, Erastin increased the level of MDA, the end product of lipid peroxidation, which was also impaired by downregulated ZEB1 (Fig. 5F).

### KLF5 is correlated with ZEB1 and HMOX1 in PDAC tissues

**Fig. 6:**
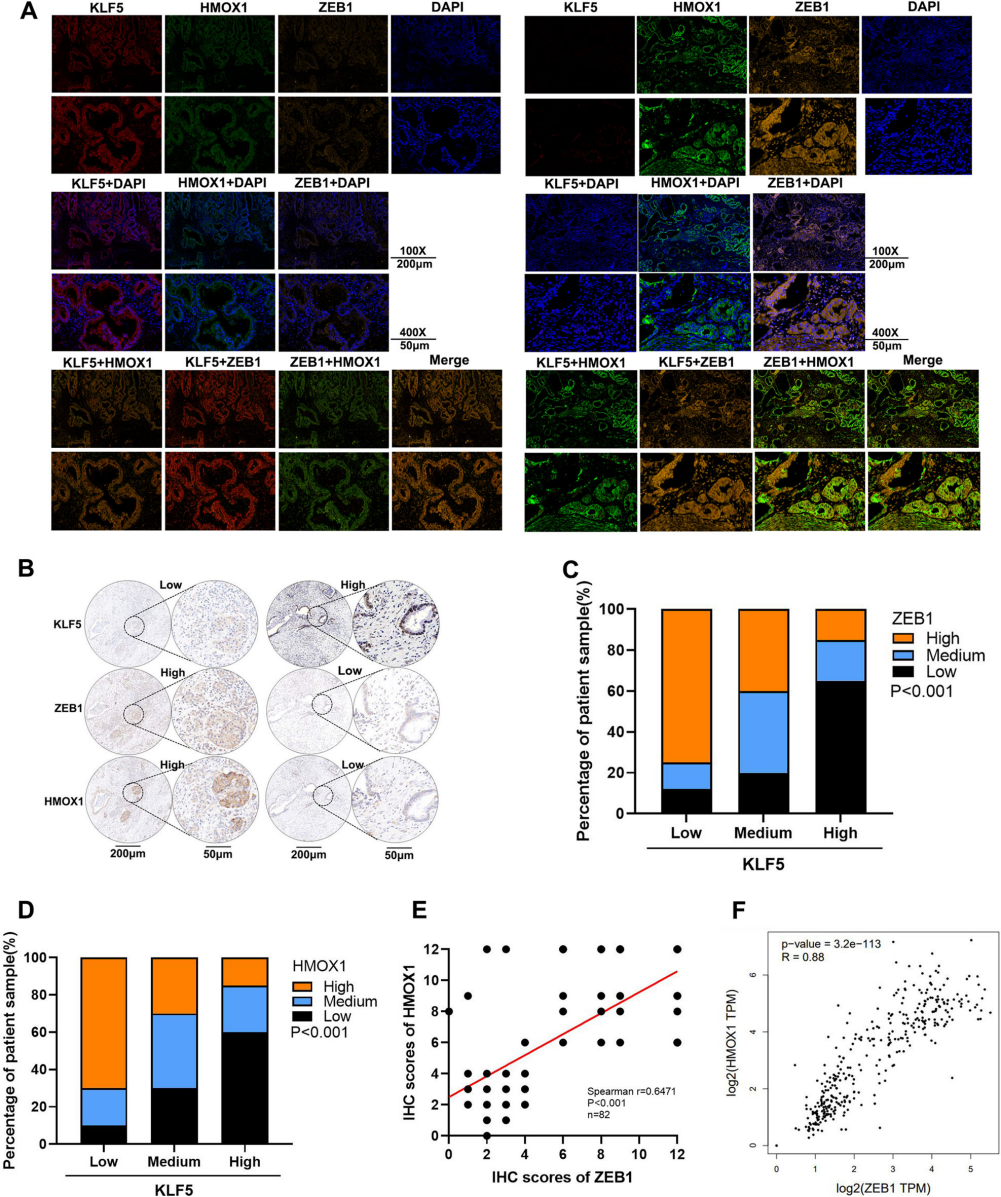
KLF5 is correlated with ZEB1 and HMOX1 in PDAC tissues. **A** Immunofluorescence of KLF5, ZEB1, and HMOX1 in tissues of patients with PDAC. **B** IHC staining of KLF5, ZEB1, and HMOX1 in tissues of patients with PDAC. **C, D** Statistical analysis of the correlation between expression of KLF5 and ZEB1, or KLF5 and HMOX1 (*P* was obtained using Pearson’s *χ*^2^ test). **E** The standard to evaluate IHC staining is shown in Fig. S4B. The correlation between the expression of ZEB1 and HMOX1 was analyzed (*n* = 82, Spearman *r* = 0.6471, *P* < 0.001). **F** ZEB1 was positively correlated with HMOX1 in the GEPIA dataset.

We evaluated the relationship between KLF5, ZEB1, and HMOX1 expression in tissues from pancreatic cancer patients. KLF5, ZEB1, and HMOX1 were labeled with different fluorescent signals. When KLF5 was low, ZEB1 and HMOX1 were highly expressed. The opposite result was also found (Fig. 6A). These results were validated by IHC staining (Fig. 6B). The IHC results (Fig. S4A, B) revealed that KLF5 was negatively correlated with ZEB1 and HMOX1 (Fig. 6C, D). We verified the correlation between ZEB1 and HMOX1 expression. IHC staining showed that ZEB1 was positively correlated with HMOX1 in PDAC tissues (Fig. 6E). TCGA and GTEx data also showed that ZEB1 was positively correlated with HMOX1 (Fig. 6F).

### KLF5 inhibitor sensitizes pancreatic xenograft tumors to oxaliplatin

**Fig. 7:**
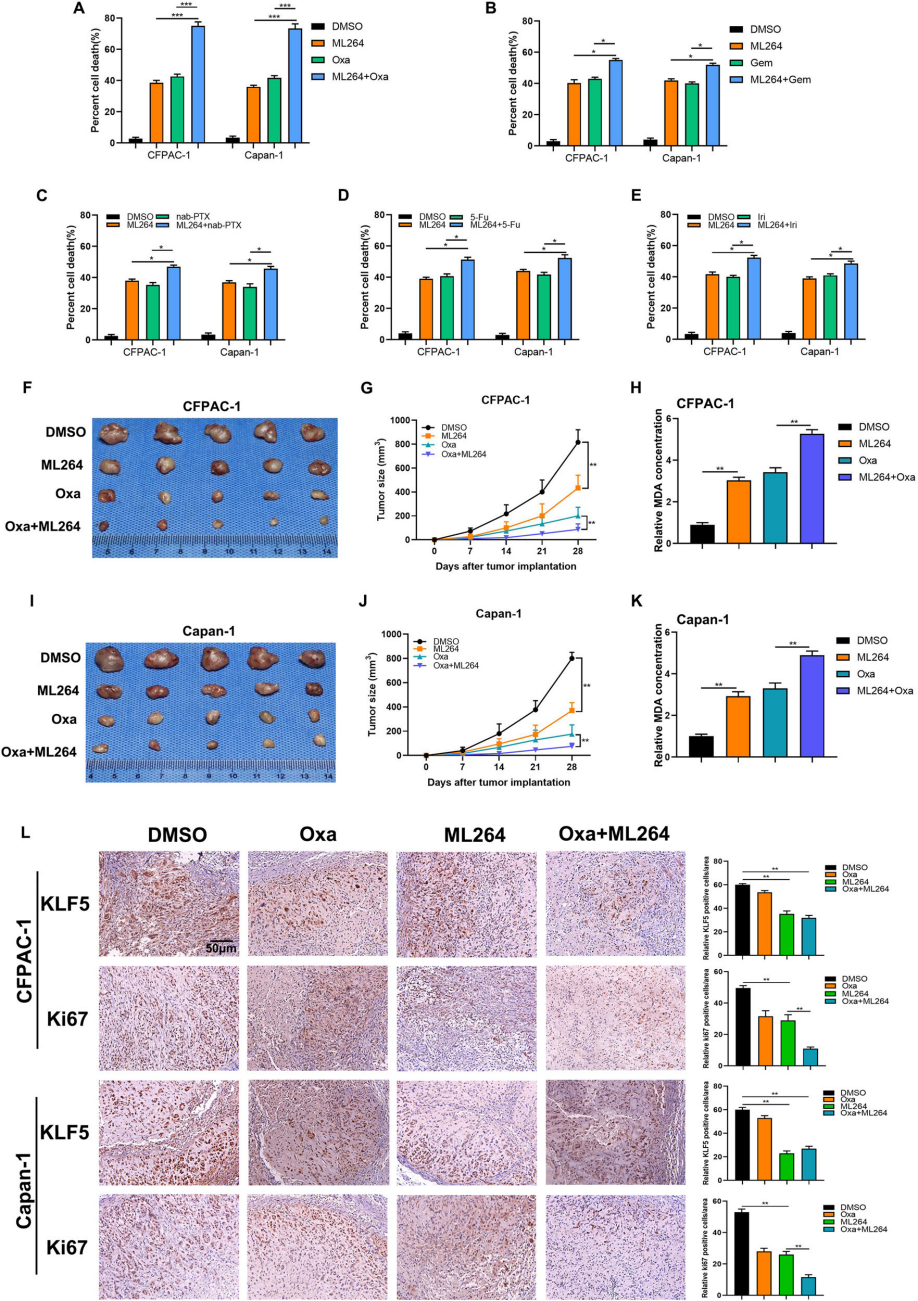
Inhibitor of KLF5 facilitates the cytotoxic effect of oxaliplatin. **A**–**E** PDAC cells were treated with ML264 in the presence or absence of oxaliplatin (Oxa) or gemcitabine (Gem), or nab-paclitaxel (nab-PTX), or 5-FU, or irinotecan (Iri) for 48 h, and cell death was detected by PI labeling. CFPAC-1 and Capan-1 cells were subcutaneously inoculated in nude mice. The mice were divided into NC, ML264, Oxa, and ML264+Oxa groups. **F**, **I** Tumor size was measured using vernier calipers (**P < 0.01). **G**, **J** Tumor growth curves depicted the tumor volumes. **H**, **K** MDA levels of the indicated tumors were detected. **L** Expression of KLF5 and the proliferation marker Ki-67 were detected in tumor tissue sections from the xenografts using IHC staining (*n* = 5, scale bar, 50 μm).

We evaluated the clinical significance of ferroptosis in PDAC. It has been established that platinum compounds promote ferroptosis through depleting glutathione (GSH) [46]. Ferroptosis inducer Erastin has been reported to facilitate the cytocidal effect of cisplatin and gemcitabine in PDAC cells [47]. At present, oxaliplatin, 5-FU, irinotecan, gemcitabine, and nab-paclitaxel are the cornerstone of chemotherapy for pancreatic cancer. KLF5 inhibitor ML264 sensitized PDAC cells to RSL3 (Fig. S5A) and Erastin (Fig. S5B). ML264 combined with oxaliplatin had the most significant combined cytotoxic effect (Fig. 7A–E). We further investigated whether KLF5 inhibitors could enhance sensitivity to oxaliplatin using cell proliferation and colony formation assays. CCK-8 assay results showed that treatment with either oxaliplatin or ML264 alone reduced cell growth, while their combination led to an even greater decrease in cell viability (Fig. S5C). Similarly, both oxaliplatin and ML264 individually suppressed colony formation in CFPAC-1 and Capan-1 cells, with ML264 significantly enhancing the inhibitory effect of oxaliplatin (Fig. S5D, E). We verified the effect of the combination of ML264 and oxaliplatin in vivo. The pancreatic xenograft tumors using CFPAC-1 cells revealed that a combination of ML264 and oxaliplatin inhibited tumor growth rate and size more than either drug alone (Fig. 7F, G) and resulted in the highest level of MDA (Fig. 7H). The results were confirmed in pancreatic xenograft tumors using Capan-1 cells (Fig. 7I–K). IHC staining using antibodies against KLF5 and the proliferation marker Ki-67 showed that ML264 combined with oxaliplatin resulted in greater suppression of Ki-67 staining than either drug alone (Fig. 7L). In PDX mouse models, the combination of oxaliplatin and ML264 significantly reduced tumor growth rate and size while increasing MDA levels compared to monotherapy (Fig. S5F, G). IHC staining with Ki-67 antibodies demonstrated that ML264 combined with oxaliplatin resulted in greater suppression of Ki-67 staining than either drug alone (Fig. S5H). These findings suggest that ML264 significantly enhances the cytotoxic effects of oxaliplatin. Furthermore, we investigated the expression of KLF5 in oxaliplatin-resistant (Oxa-R) cells. Previously established Oxa-R cell lines were revived and confirmed for drug resistance through cytotoxicity assays (Fig. S6A). qPCR and WB analyses revealed that KLF5 expression was upregulated in Oxa-R CFPAC-1 and Capan-1 cells (Fig. S6B). ML264 significantly increased the sensitivity of Oxa-R cells to oxaliplatin (Fig. S6C). CCK-8 assay results showed that both oxaliplatin and ML264 individually suppressed cell growth, with their combination further decreasing cell viability (Fig. S6D). Similarly, both agents alone inhibited colony formation in CFPAC-1 and Capan-1 cells, and ML264 significantly enhanced oxaliplatin’s inhibitory effects (Fig. S6E, F). These results indicate that KLF5 inhibition may help overcome acquired resistance to oxaliplatin.

There is no doubt that inducing ferroptosis can reduce cell viability and increase sensitivity to platinum drugs. In addition to cell activity, we also explored other pathways of ferroptosis, including ketogenesis, stemness, and iron homeostasis. Cells incubated with βHB can simulate a ketogenic diet in mice. The results show that the added βHB (10 mM) induced an increase in lipid peroxidation and MDA, which could be reversed by Fer-1 in CFAPC-1 and Capan-1 cells (Fig. S7A, B). The results indicated ketogenesis may promote ferroptosis in pancreatic cancer. Correlation analysis using TCGA and GTEx data showed that there is a positive correlation between the expression of KLF5 and stemness markers PROM1, ABCG2, SOX2, and CD44 (Fig. S7C), and there is a negative correlation between the expression of KLF5 and differentiated cell markers ATP4B and MUC6 (Fig. S7D). Moreover, the results of the spheres formation experiment suggested that inhibition of KLF5 decreases the number of formed spheres (Fig. S7E). These results indicated the stemness of pancreatic cancer may be weakened by downregulating KLF5. Our previous results in Fig. 2 showed that the suppression of KLF5 could induce ferroptosis in pancreatic cancer. Therefore, we speculate that pancreatic cancer cells with reduced stemness may be more prone to ferroptosis. We also used the probe Ferroorange to detect the level of ferrous ions and a cellular iron detection kit to detect the level of total iron. We observed that inhibition of KLF5 increased the level of ferrous ion (Fig. S7F) and total iron (Fig. S7G), which could be reversed by Fer-1. These results indicated that inhibition of KLF5 could disrupt the intracellular iron homeostasis, increase the iron load, and induce ferroptosis.

## Discussion

PDAC remains one of the most fatal malignancies, with an overall 5-year survival rate of only ~11% [48]. Surgery and systemic chemotherapy remain the mainstay of treatment of this disease [49, 50]. Unfortunately, long-term survival for PDAC patients is rare due to innate insensitivity and rapidly developing chemoresistance. Consequently, there is an urgency to explore novel approaches to kill pancreatic cancer cells and develop new treatment strategies effectively.

Ferroptosis as a distinctive form of cell death has attracted much interest in cancer research. The unique metabolism of tumor cells renders them intrinsically susceptible to ferroptosis, thereby showing defects that could be therapeutically targetable in some cancer types [51–53]. Ferroptosis has also been induced by several cancer therapies, including chemotherapy [54], immunotherapy [29], and radiotherapy [55]. It was also reported that pharmacological inhibition of system xCT facilitated the cytotoxic effects of both cisplatin and gemcitabine in PDAC cell lines [47]. Thus, ferroptosis inducers have potential in pancreatic cancer therapy, especially in combination with traditional therapies.

In this study, we discovered that KLF5 was highly expressed in PDAC and closely associated with cancer progression. KLF5 is mainly expressed in cancer cells among the multiple cellular components of tumor tissue. TMAs indicated that expression of KLF5 was upregulated in PDAC samples compared with adjacent normal tissues. High expression of KLF5 correlated with poor OS in PDAC patients. High-throughput sequencing showed that inhibition of KLF5 promoted ferroptosis in PDAC cells. Further results demonstrated that suppression of KLF5 potentiated ferroptosis. To clarify the inherent mechanisms, we comprehensively analyzed the FerrDb database and gene expression profile in TCGA and high-throughput screening. Subsequent qPCR and WB identified HMOX1 as the effector gene of KLF5-mediated ferroptosis. HMOX1 catalyzes the degradation of heme to ferrous iron, CO, and biliverdin. Excessive activation of HMOX1 potentiates ferroptosis by increasing the labile iron pool [56]. The atypical feature of noncanonical ferroptosis is increased intracellular labile ferrous iron upon excessive upregulation of HMOX1, which is sufficient to trigger ferroptosis [57]. GSK-J4 and donafenib synergistically facilitate the expression of HMOX1 and enhance intracellular ferrous iron level, finally causing ferroptosis in liver cancer [58]. HMOX1 can also function in a cytoprotective way, perhaps depending on the level of activation [59]. The cytoprotective effect of HMOX1 is due to its antioxidant activity, while its cytotoxic effect is attributed to the increased production of ferrous iron. Consequently, extreme activation of HMOX1 could be cytotoxic, while mild upregulation could be protective [60]. We confirmed that KLF5 inhibited the transcription of HMOX1, and downregulation of HMOX1 could powerfully mitigate decreased KLF5-induced ferroptosis.

Due to KLF5 downregulating HMOX1 at the transcriptional level, we first assumed that HMOX1 was the target gene for transcription factor KLF5. However, KLF5 could not bind to the promoter site of HMOX1. Thus, KLF5 might mediate the transcription of a TF, which could bind to the HMOX1 promotor to enhance HMOX1 transcription. We intersected the predicted transcription factors of HMOX1 with differentially expressed genes in the RNA sequence. Further qPCR and WB identified ZEB1 as a direct transcription factor of HMOX1, and KLF5 inhibited the expression of ZEB1, which is a driver of epithelial-to-mesenchymal transition and a lipogenic factor [61]. Synthesis of phospholipids containing polyunsaturated fatty acids is increased in cancer cells in the mesenchymal state, probably owing to the core position of ZEB1 in lipid metabolism [61]. O-GlcNAcylation of ZEB1 potentiates mesenchymal PDAC cell ferroptosis [62]. We verified that KLF5 regulated HMOX1 via ZEB1. However, one shortcoming of our study was that we did not reveal how KLF5 regulated ZEB1. It has been reported that KLF5 facilitates miRNA 200 transcription to inhibit the expression of ZEB1 [63]. Thus, we speculated that KLF5 repressed ZEB1 via one miRNA or transcription factor.

ZEB1 is a key molecule regulating epithelial-mesenchymal transition (EMT). The EMT is considered a cellular process to induce cell stemness, which causes tumor metastasis and treatment resistance, for instance, ZEB1, SNAI1, and TWIST1 [64]. Likewise, EMT is associated with inducing ferroptosis. Highly metastatic cells are often more sensitive to ferroptosis [51]. Metastasis key molecule ZEB1 is believed to mediate EMT-associated ferroptosis sensitivity in pancreatic cancer cells by regulating lipogenic enzyme expression and phospholipid composition [65]. In addition, Drug-resistant cancer cells, especially those in a mesenchymal state and prone to metastasis, are susceptible to ferroptosis, which could bring a promising strategy for drug-resistant cancer treatment [66]. Mechanically, drug-resistant pancreatic cancer cells have vigorous fatty acid metabolism, which is an important part of regulating ferroptosis. Inducing ferroptosis due to an imbalance in fatty acid metabolism can make drug-resistant cells more sensitive to chemotherapeutic drugs [67].

**Fig. 8:**
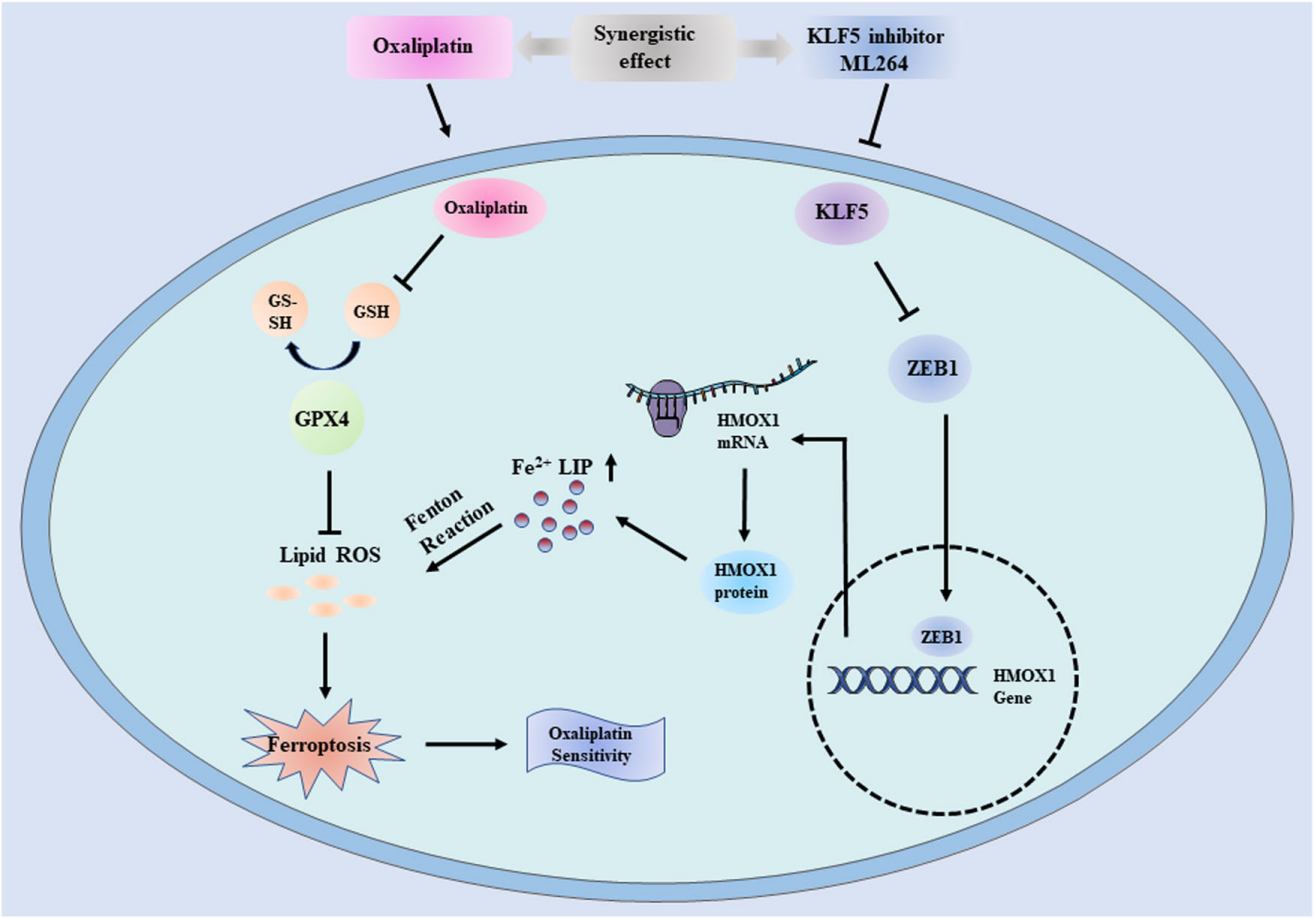
Schematic representation of the model. Graphical abstract indicating the mechanism of KLF5-mediated ferroptosis via ZEB1/HMOX1 axis in pancreatic cancer cells and the role of ML264 in sensitizing oxaliplatin based on its significant facilitation of ferroptosis.

At present, the main chemotherapeutic drugs used in pancreatic cancer include oxaliplatin, 5-FU, irinotecan, gemcitabine, and nab-paclitaxel. Platinum compounds have a stronger affinity for thiol-rich molecules. GSH is one of the most abundant and critical non-protein thiols in cells. Most intracellular cisplatin in the cytoplasm is conjugated to GSH [46]. Thus, cisplatin can induce ferroptosis through depleting GSH and subsequently inactivating glutathione peroxidase, which is similar to ferroptosis activator Erastin. It was also reported that drug resistance was involved in platinum-induced apoptosis, but not platinum-induced ferroptosis. Thus, ferroptosis combined with traditional chemotherapy may be a better strategy for PDAC. Our results demonstrated that inhibition of KLF5 could induce ferroptosis and KLF5 inhibitor combined with oxaliplatin, rather than the other four drugs, had the greatest synergistic killing effect in vitro. Compared to ML264 or oxaliplatin alone, ML264 combined with oxaliplatin exerted a greater inhibitory effect on tumor growth in vivo. Collectively, our results demonstrated for the first time that inhibition of KLF5 triggers ferroptosis in PDAC cells by activating the ZEB1/HMOX1 axis. Oxaliplatin, as a ferroptosis activator, combined with compounds inducing ferroptosis showed a powerful cytocidal effect on PDAC cells (Fig. 8). Thus, the combination of activation of the KLF5/ZEB1/HMOX1 axis and oxaliplatin may provide a potential therapeutic strategy for pancreatic cancer.

## Supplementary information

Supplementary materials

## Data Availability

The supporting data of this study are available from the corresponding author upon reasonable request.
