# Supplementary material for: Inhibition of KLF5 promotes ferroptosis via the ZEB1/HMOX1 axis to enhance sensitivity to oxaliplatin in cancer cells
Source: Cell Death Dis. 2025 Jan 18;16(1):28. doi: 10.1038/s41419-025-07330-8 (PMC11743205; doi:10.1038/s41419-025-07330-8)
Supplement: Supplementary file 1 — Supplementary materials [file 41419_2025_7330_MOESM1_ESM.pdf]

Supplemental Figures and Legends

A

KLFs	OS(P-value)	DFS(P-value)
KLF1	0.4	0.88
KLF2	0.67	0.45
KLF3	0.0013	0.097
KLF4	0.29	0.26
KLF5	0.01	0.023
KLF6	0.041	0.15
KLF7	0.48	0.024
KLF8	0.64	0.46
KLF9	0.34	0.41
KLF10	0.22	0.22
KLF11	0.087	0.14
KLF12	0.31	0.4
KLF13	0.65	0.47
KLF14	0.43	0.56
KLF15	0.042	0.041
KLF16	0.094	0.33
KLF17	0.053	0.11

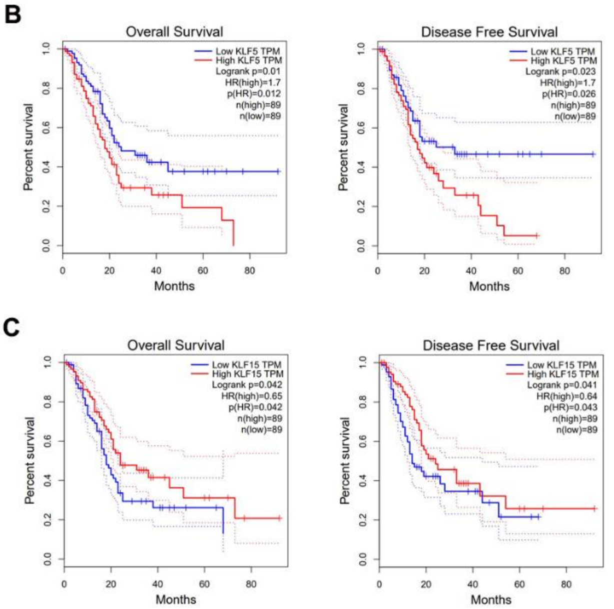

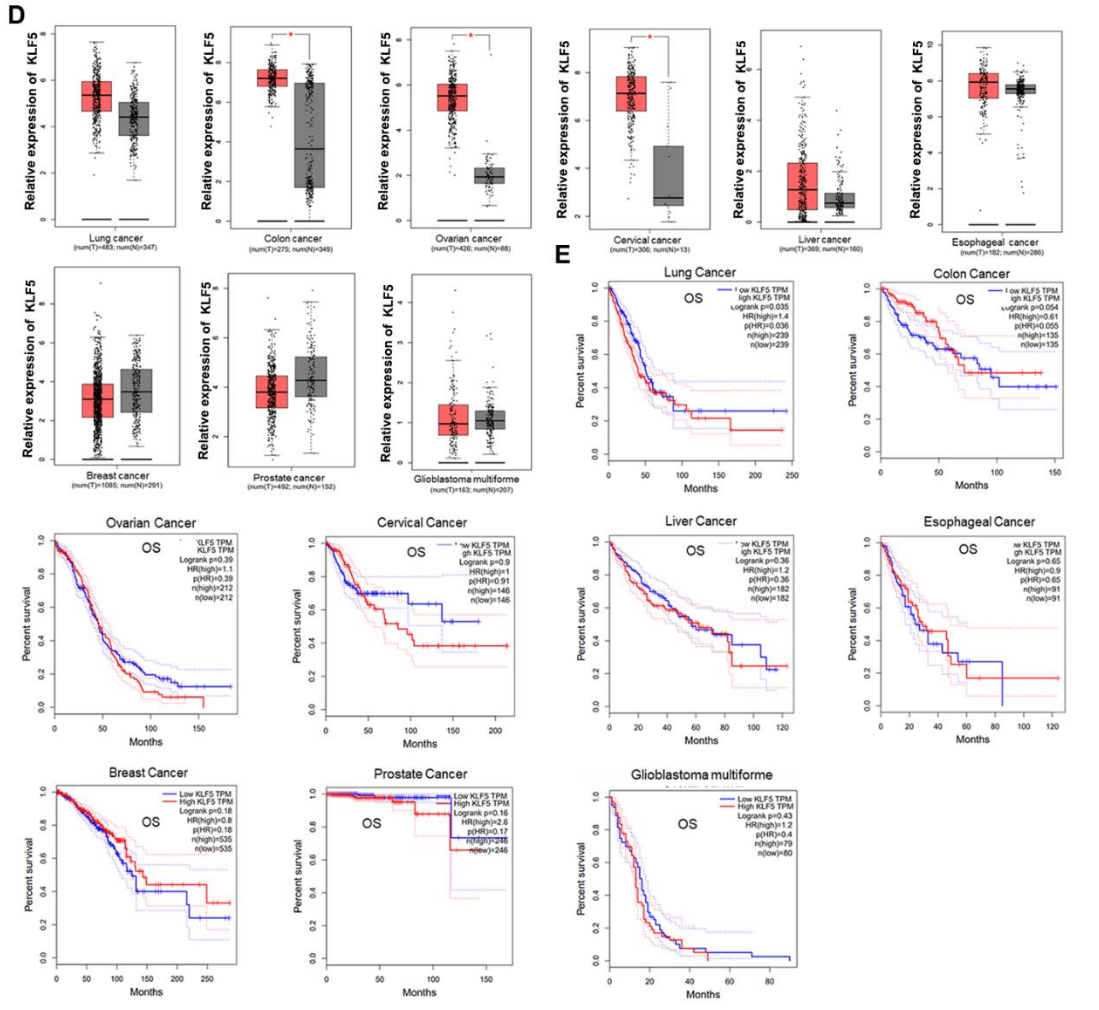

Fig. S1 Correlation between KLF family and prognosis of patients with pancreatic

cancer. A OS and DFS of PDAC patients were evaluated using Kaplan–Meier analysis according to the mRNA expression of 17 members of the KLF family. **B** OS and DFS of PDAC patients were evaluated and depicted according to mRNA expression of KLF5. **C** OS and DFS of PDAC patients were evaluated and depicted according to mRNA expression of KLF15. **D** KLF5 mRNA expression levels in cancers from TCGA and para-cancerous tissue matching TCGA normal and GTEx data (*$P < 0.05$). **E** OS of patients were evaluated according to mRNA expression of KLF5.

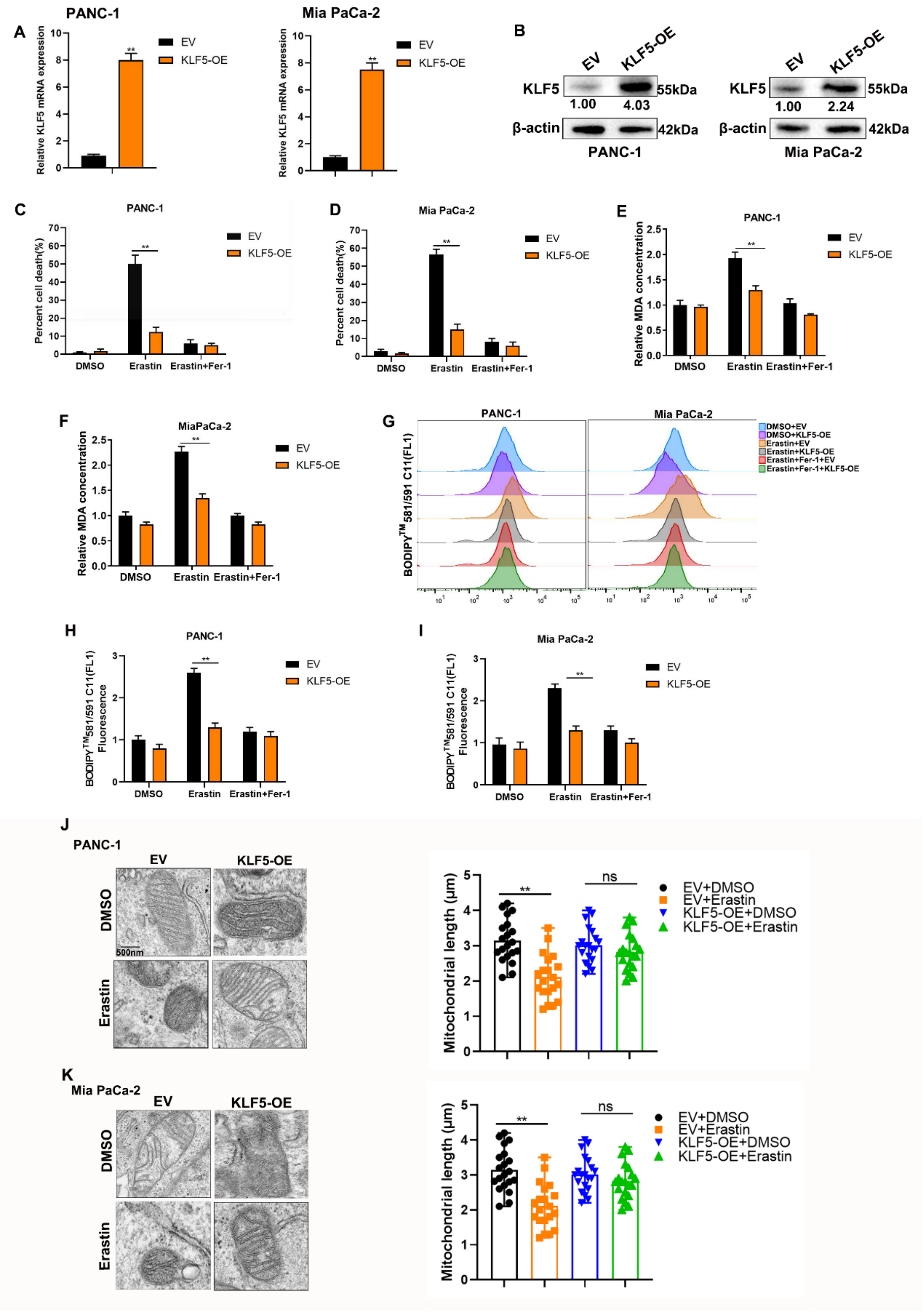

Fig. S2 Overexpression of KLF5 inhibits ferroptosis. A qRT-PCR evaluated overexpression of KLF5. **B** WB evaluated overexpression of KLF5. **C, D** KLF5-

overexpressing PANC-1 and Mia PaCa-2 cells were treated with 2.5 μmol/L Erastin and/or Fer-1 (2 μmol/L) for 16 h. Cell death was detected using PI labeling. **E, F** PANC-1 and Mia PaCa-2 cells were subjected to the same treatment and MDA was detected. **G–I** PANC-1 and Mia PaCa-2 cells were subjected to the same treatment and lipid peroxidation was analyzed using BODIPY 581/591 C11. **J, K** TEM was performed on PANC-1 and Mia PaCa-2 cells treated by Erastin.

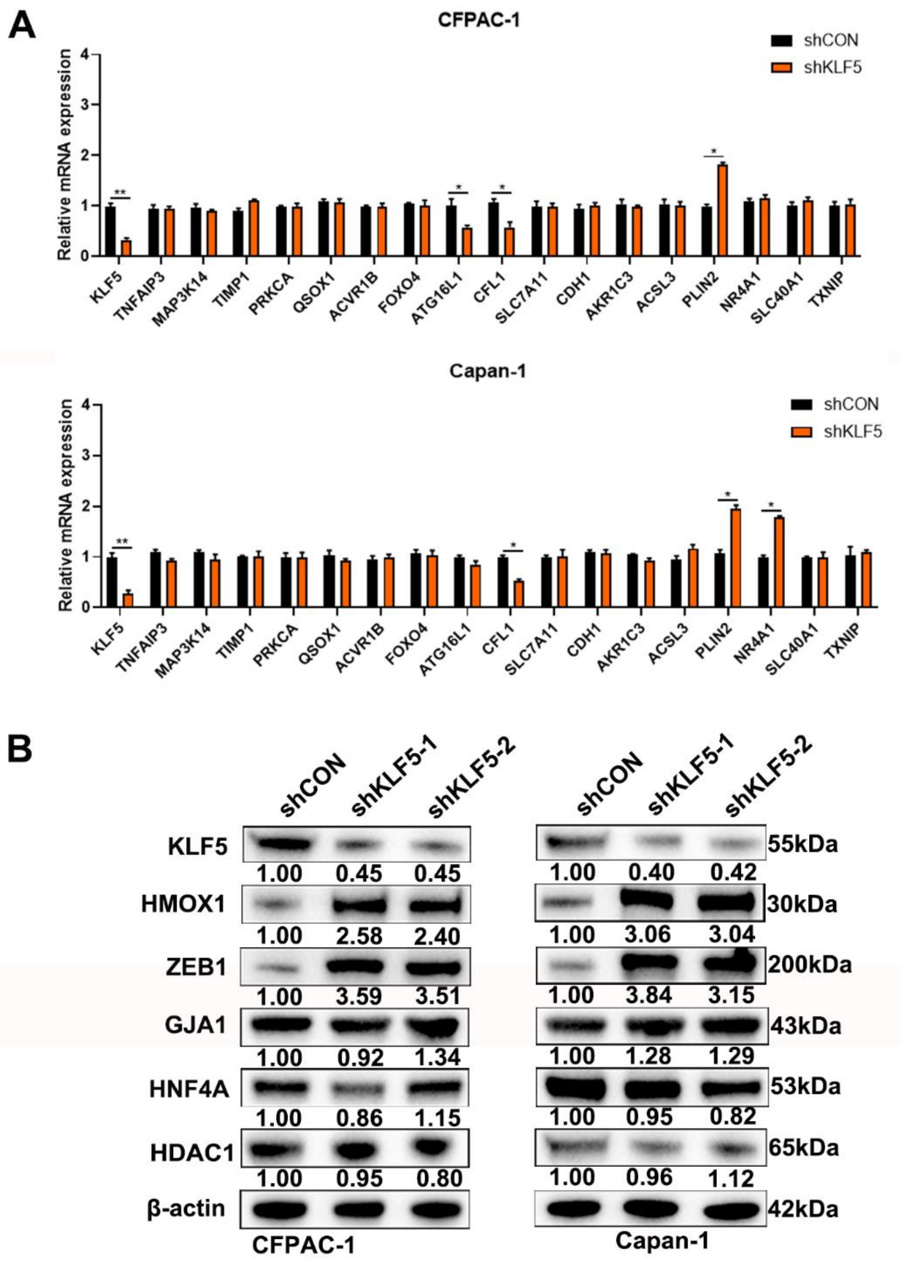

Fig. S3 KLF5 downregulation causes changes in mRNA and proteins. A qPCR analyzed the changes in the mRNA caused by KLF5 downregulation. **B** WB analyzed the changes in the proteins caused by KLF5 downregulation.

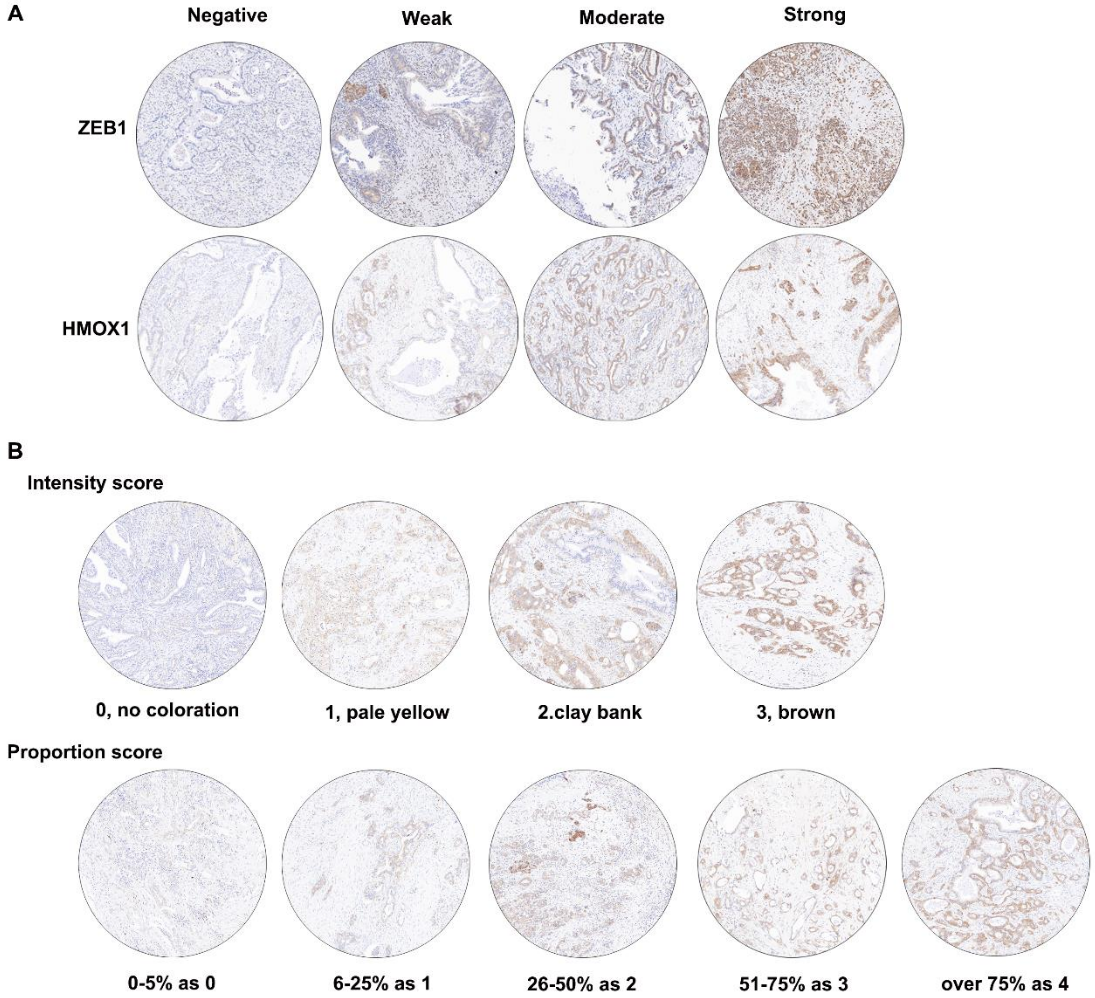

Fig. S4 ZEB1 positively correlated with HMOX1 expression in PDAC patients. A IHC staining of ZEB1 and HMOX1 in tissues from PDAC patients. **B** Micrographs depicting intensity score and proportion score of ZEB1 and HMOX1.

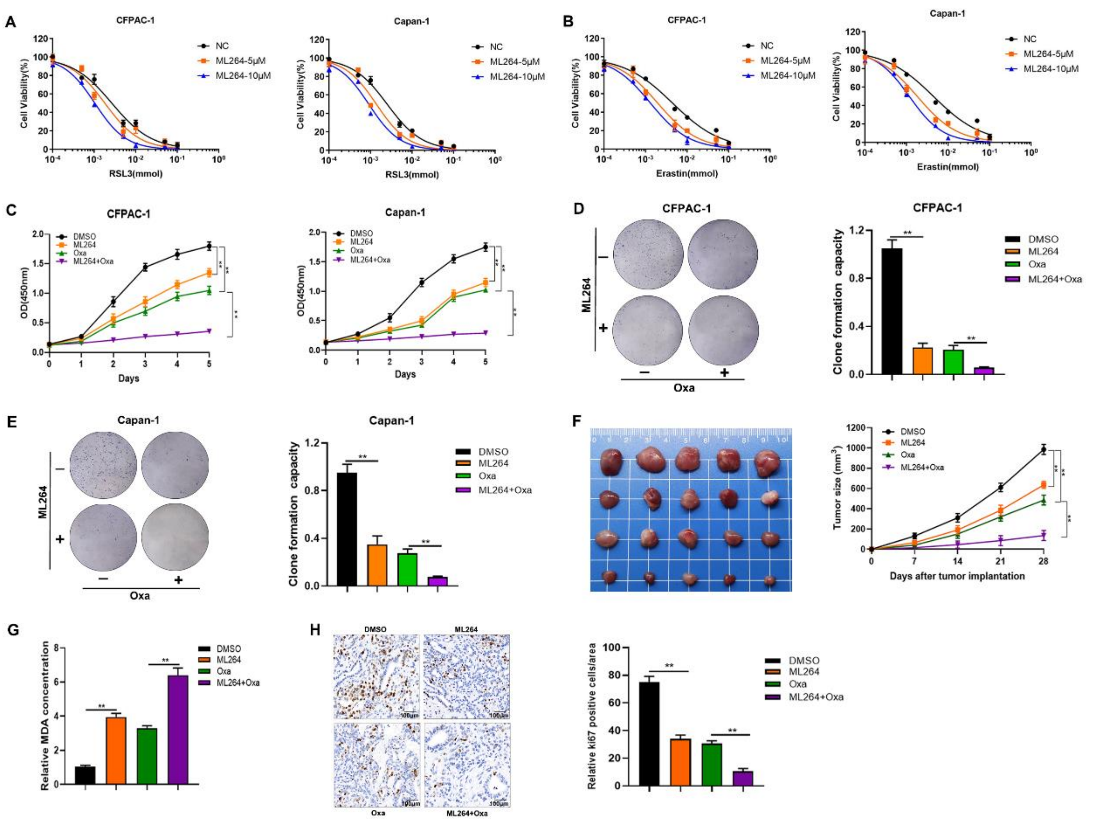

Fig. S5 ML264 enhances the cytotoxic effect of oxaliplatin. A ML264 increased sensitivity to RSL3 in PDAC cells. **B** ML264 increased sensitivity to Erastin in PDAC cells. **C** Cell proliferation in CFPAC-1 and Capan-1 cells treated with oxaliplatin and ML264 was assessed using the CCK-8 assay. **D, E** The influence of oxaliplatin and ML264 on CFPAC-1 and Capan-1 cells were evaluated through colony formation assays. **F** Mice were randomly assigned to four groups: DMSO, oxaliplatin, ML264, and oxaliplatin + ML264. Treatment was administered as described in the Methods section, and tumor sizes were measured using Vernier calipers. **G** MDA level in tumor samples. **H** Expression of the proliferation marker Ki-67 in tumor tissue sections from xenografts was analyzed by IHC staining (n=5, scale bar, 100 μm). (n=5, scale bar, 100 μm).

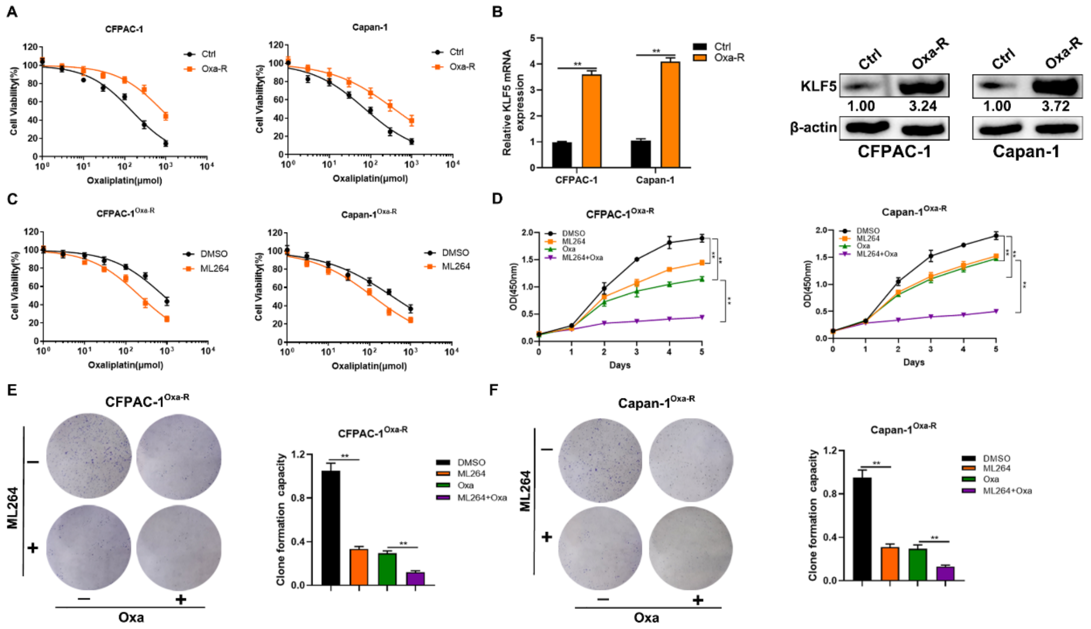

Fig. S6 Inhibition of KLF5 alleviates oxaliplatin-induced resistance. A Dose-response curves for oxaliplatin in control and Oxa-R CFPAC-1 and Capan-1 cells. **B** mRNA and protein levels of KLF5 in control and Oxa-R cells. **C** Dose-response curves for combined ML264 and oxaliplatin treatment in Oxa-R cells. **D** Cell proliferation in Oxa-R cells treated with oxaliplatin and ML264 was evaluated using the CCK-8 assay. **E, F** Effects of oxaliplatin and ML264 on Oxa-R cells were assessed by colony formation assays.

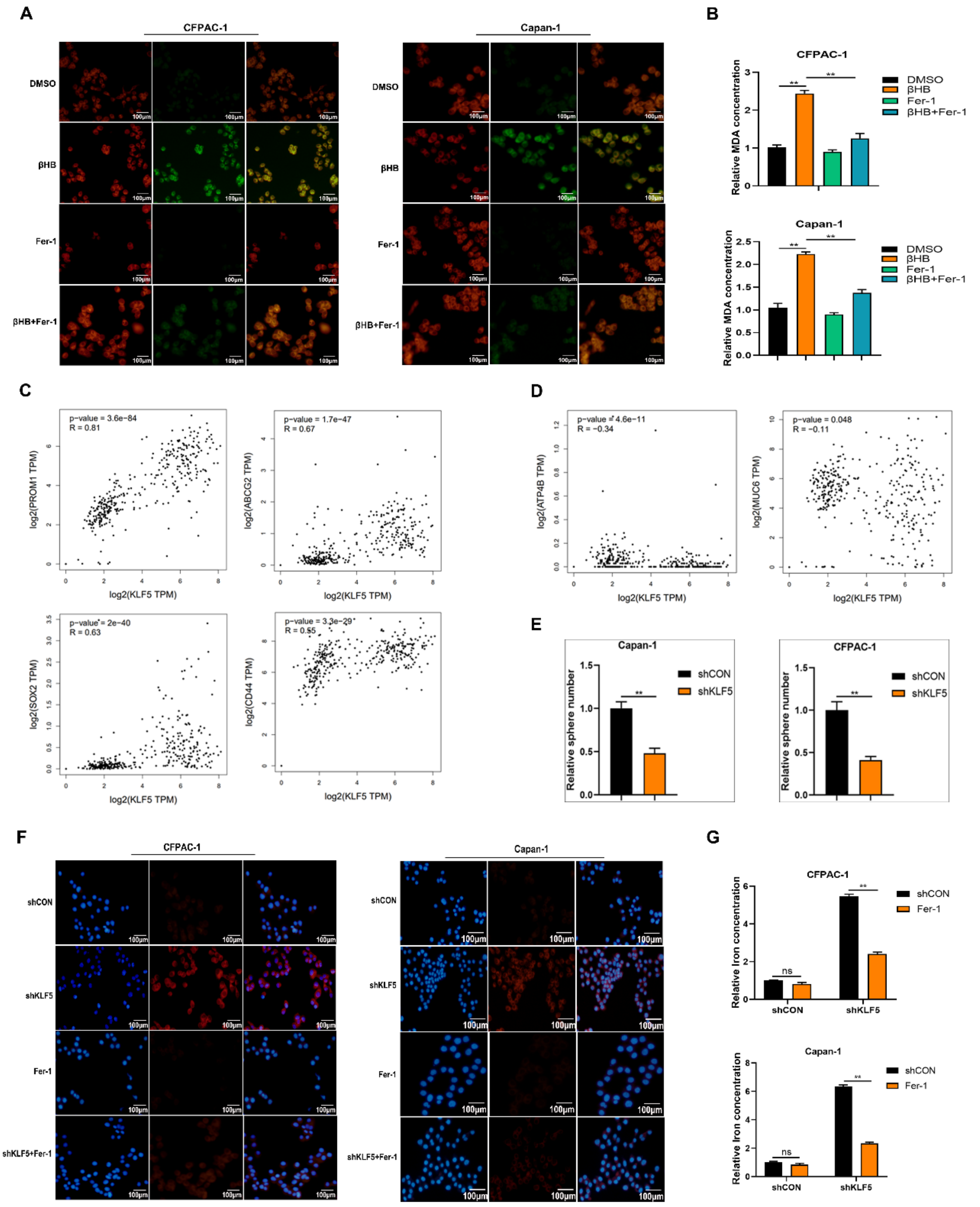

Fig. S7 Ferroptosis is associated with ketogenesis, stemness, and iron homeostasis.

A Confocal imaging revealed the effect of βHB (10mM) on lipid peroxidation of CFPAC-1 and Capan-1 cells. **B** MDA level was analyzed in βHB (10mM)-treated CFPAC-1 and Capan-1 cells in the presence or absence of 2 μmol/L Fer-1. **C** Correlation analysis between KLF5 and stemness markers PROM1, ABCG2, SOX2,

and CD44. **D** Correlation analysis between KLF5 and differentiated cell markers ATP4B and MUC6. **E** The cell stemness mediated by KLF5 was evaluated using a sphere formation assay. **F** Confocal imaging revealed the effect of inhibition of KLF5 on the level of ferrous ions in CFPAC-1 and Capan-1 cells. **G** The total iron level was detected in KLF5-silenced CFPAC-1 and Capan-1 cells in the presence or absence of 2 μmol/L Fer-1.

Supplementary Table 1 Primer sequences of qPCR used in this study

Genes	Sequence (5' -> 3')
HNF4A forward	CACGGGCAAACACTACGGT
HNF4A reverse	TTGACCTTCGAGTGCTGATCC
TXN forward	GTGAAGCAGATCGAGAGCAAG
TXN reverse	CGTGGCTGAGAAGTCAACTACTA
CTSB forward	GAGCTGGTCAACTATGTCAACA
CTSB reverse	GCTCATGTCCACGTTGTAGAAGT
TSC22D3 forward	AACACCGAAATGTATCAGACCC
TSC22D3 reverse	TGTCCAGCTTAACGGAAACCA
DUSP1 forward	AGTACCCCACTCTACGATCAGG
DUSP1 reverse	GAAGCGTGATACGCACTGC
SMPD1 forward	CTGTCTGACTCTCGGGTTCTC
SMPD1 reverse	CTATGCGATGTAACCTGGCAG
RRM2 forward	CACGGAGCCGAAAACTAAAGC
RRM2 reverse	TCTGCCTTCTTATACATCTGCCA
MUC1 forward	TGCCGCCGAAAGAACTACG
MUC1 reverse	TGGGGTACTCGCTCATAGGAT
HERPUD1 forward	ATGGAGTCCGAGACCGAAC
HERPUD1 reverse	TTGGTGATCCAACAACAGCTT
ACSL1 forward	CCATGAGCTGTTCCGGTATTT
ACSL1 reverse	CCGAAGCCCATAAGCGTGTT
DAZAP1 forward	AGACCGCACACGCTAGATG
DAZAP1 reverse	TGACCACTCCGAACTTCTTGA
NCF2 forward	CCCACTCCCGGATTTGCTTC
NCF2 reverse	GTCTCGGTTAATGCTTCTGGTAA
EPAS1 forward	CGGAGGTGTTCTATGAGCTGG
EPAS1 reverse	AGCTTGTGTGTTCGCAGGAA
HMGB1 forward	TATGGCAAAAGCGGACAAGG
HMGB1 reverse	CTTCGCAACATCACCAATGGA
GPX2 forward	GGTAGATTTCAATACGTTCCGGG
GPX2 reverse	TGACAGTTCTCCTGATGTCCAAA
GABARAPL1 forward	ATGAAGTTCCAGTACAAGGAGGA
GABARAPL1 reverse	GCTTTTGGAGCCTTCTCTACAAT
TGFB1 forward	GGCCAGATCCTGTCCAAGC
TGFB1 reverse	GTGGGTTTCCACCATTAGCAC
BNIP3 forward	CAGGGCTCCTGGGTAGAACT
BNIP3 reverse	CTACTCCGTCCAGACTCATGC
LIFR forward	TGGAACGACAGGGGTTCAGT
LIFR reverse	GAGTTGTGTTGTGGGTCACTAA
GJA1 forward	GGTGACTGGAGCGCCTTAG
GJA1 reverse	GCGCACATGAGAGATTGGGA
ZEB1 forward	GATGATGAATGCGAGTCAGATGC
ZEB1 reverse	ACAGCAGTGTCTTGTTGTTGT

HMOX1 forward	AAGACTGCGTTCCTGCTCAAC
HMOX1 reverse	AAAGCCCTACAGCAACTGTCG
β-actin forward	GAGACCTTCAACACCCCAGC
β-actin reverse	GATAGCACAGCCTGGATAGCA
KLF5 forward	ACACCAGACCGCAGCTCCA
KLF5 reverse	TCCATTGCTGCTGTCTGATTTGTAG
HDAC1 forward	CTACTACGACGGGGATGTTGG
HDAC1 reverse	GAGTCATGCGGATTCGGTGAG
ZNF362 forward	CCTTGTAGTCCCCTATCCCATC
ZNF362 reverse	CAGTGGGCATACCTTACACCT
XRCC5 forward	GTGCGGTCGGGGAATAAGG
XRCC5 reverse	GGGGATTCTATACCAGGAATGGA
ZNF217 forward	AAACATGCCAACTCAATCCCTC
ZNF217 reverse	GGAATGGAACAACAGCGGT
HNRNPL forward	TACGCAGCCGACAACCAAATA
HNRNPL reverse	CTCCGGGAGTCATCCGAGT
RUNX3 forward	AGGCAATGACGAGAACTACTCC
RUNX3 reverse	CGAAGGTCGTTGAACCTGG
POU5F1 forward	CTGGGTTGATCCTCGGACCT
POU5F1 reverse	CCATCGGAGTTGCTCTCCA
GLIS2 forward	AGCTGAGTATCACCAAGCTCC
GLIS2 reverse	AGGCTGAGGTCCACGAGAG
SOX13 forward	CCAGAGGGTAATGGGTCCC
SOX13 reverse	TGGCTTCCATAGAGTTCCTTCC
TRIM28 forward	TTTCATGCGTGATAGTGGCAG
TRIM28 reverse	GCCTCTACACAGGTCTCACAC
MXD4 forward	AACAGGTCTTCACACAACGAG
MXD4 reverse	CTGCTCCTTGATGCTCAGTG
NFIL3 forward	AAAATGCAGACCGTCAAAAAGGA
NFIL3 reverse	TGACACTTCCGTTAAAGCAGAAT
TFDP1 forward	AATTGAAGCCAACGGAGAACTC
TFDP1 reverse	CGGTCTCTGAGGCGTACCA

Supplementary Table 2 Primer sequences of ChIP used in this study

KLF5-HMOX1	Sequence (5' -> 3')
ChIP forward 1	AGCCTGGGGTTGCTAAGTTC
ChIP reverse 1	GGCTCTGGGTGTGATTTTGC
ChIP forward 2	TGGCCAGACTTTGTTTCCCA
ChIP reverse 2	GTCACATTTATGCTCGGCGG
ChIP forward 3	CAGACCTGAATGTGCCTGGA
ChIP reverse 3	ATGCCAGTGTCCAAACCCAG
ChIP forward 4	CTTTATCTGCCTAGGACAACCT
ChIP reverse 4	TGAAAGGCCGACTTTAAGGG
ChIP forward 5	AGAGACAGGGTCTCCCTATGT
ChIP reverse 5	CCCAGTACTTTGGGAGGTTG
**ZEB1-HMOX1**	
ChIP forward 6	GCCACCATGCCCAGCTAATTT
ChIP reverse 6	GGAAGAGCGCTTTGAGCCCA

Figure 1

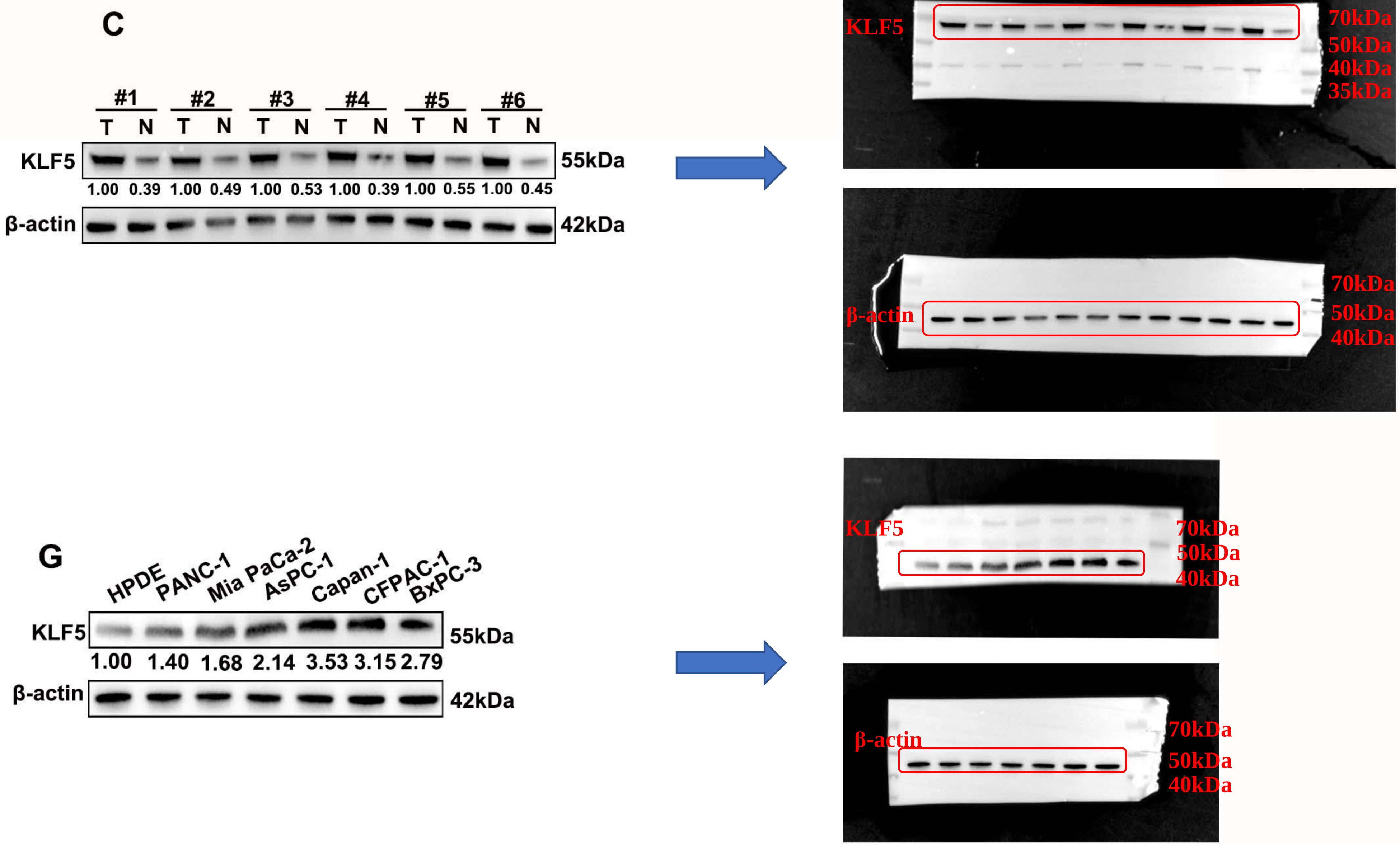

C
#1
#2
#3
#4
#5
#6
T N T N T N T N T N T N
KLF5
55kDa
1.00 0.39 1.00 0.49 1.00 0.53 1.00 0.39 1.00 0.55 1.00 0.45
β-actin
42kDa
KLF5
70kDa
50kDa
40kDa
35kDa
β-actin
70kDa
50kDa
40kDa
G
HPDE
PANC-1
Mia PaCa-2
AsPC-1
Capan-1
CFPAC-1
BxPC-3
KLF5
55kDa
1.00 1.40 1.68 2.14 3.53 3.15 2.79
β-actin
42kDa
KLF5
70kDa
50kDa
40kDa
β-actin
70kDa
50kDa
40kDa

Figure 2

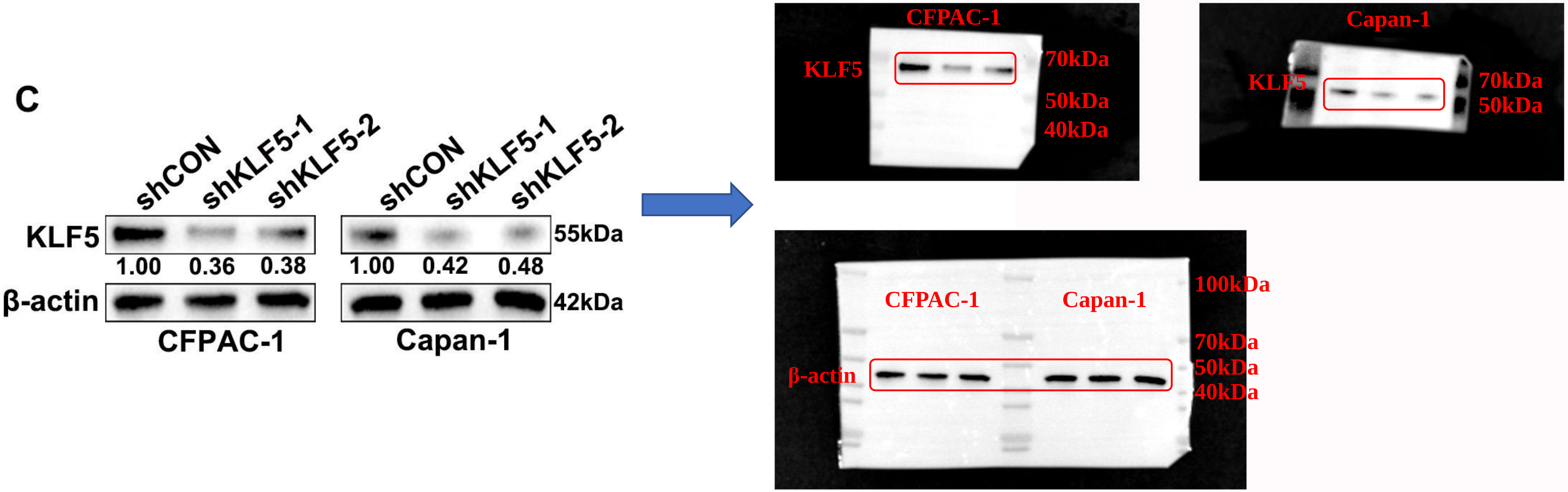
C
shCON
shKLF5-1
shKLF5-2
shCON
shKLF5-1
shKLF5-2
KLF5
1.00
0.36
0.38
1.00
0.42
0.48
55kDa
β-actin
42kDa
CFPAC-1
Capan-1
CFPAC-1
KLF5
70kDa
50kDa
40kDa
Capan-1
KLF5
70kDa
50kDa
100kDa
CFPAC-1
Capan-1
70kDa
50kDa
β-actin
40kDa

Figure 3

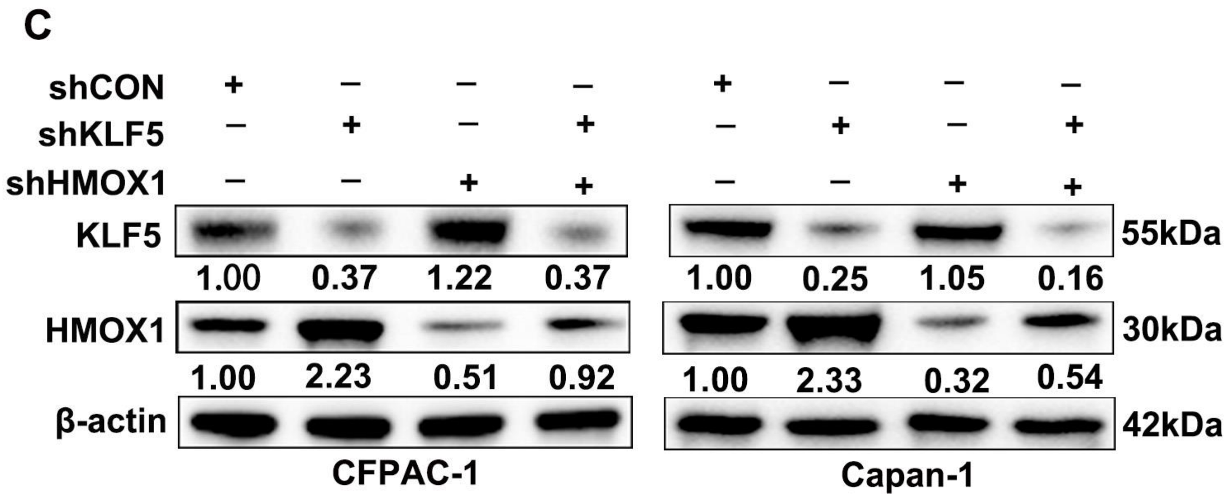
C
shCON
shKLF5
shHMOX1
KLF5
1.00 0.37 1.22 0.37
1.00 0.25 1.05 0.16
55kDa
HMOX1
1.00 2.23 0.51 0.92
1.00 2.33 0.32 0.54
30kDa
β-actin
42kDa
CFPAC-1
Capan-1

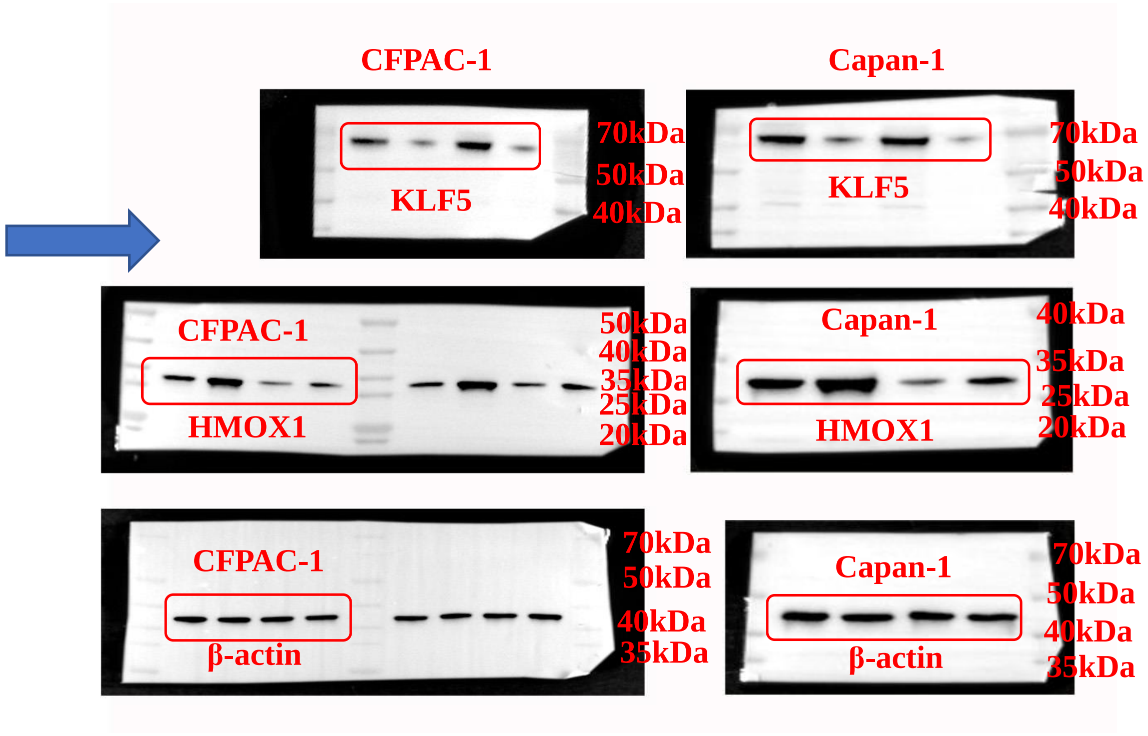
CFPAC-1
Capan-1
KLF5
70kDa
50kDa
40kDa
HMOX1
35kDa
25kDa
20kDa
β-actin

Figure 4

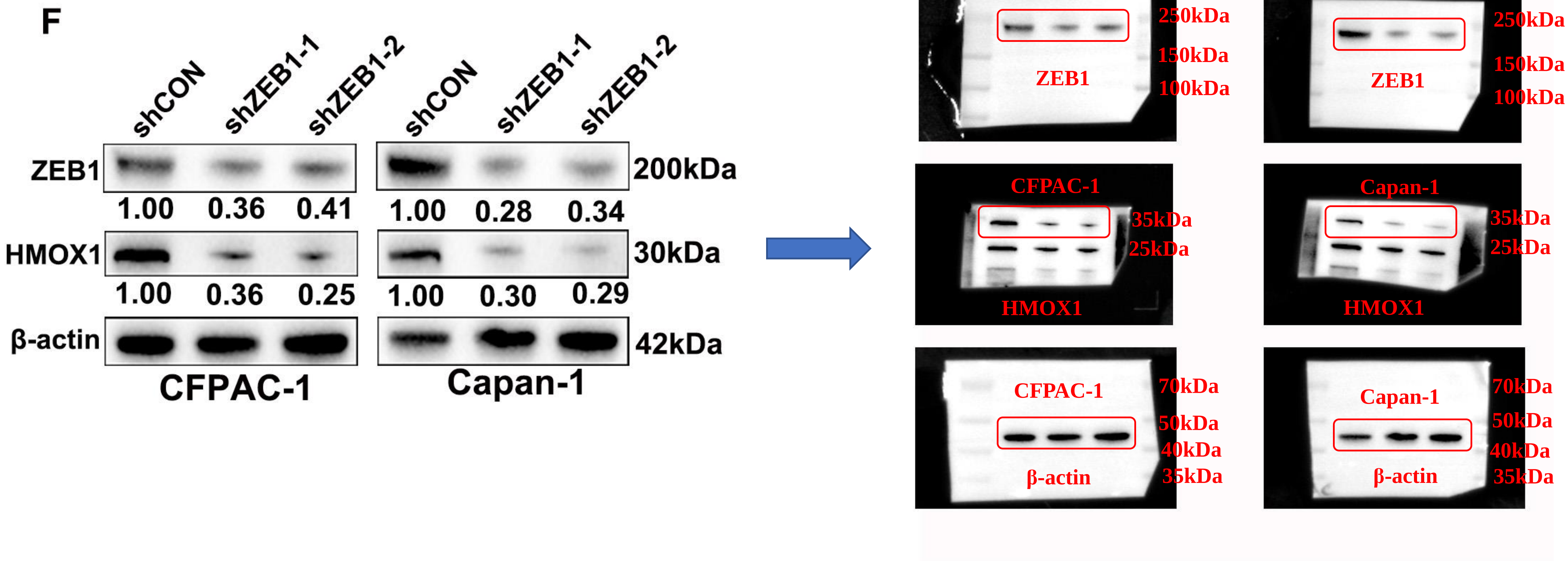
F
shCON
shZEB1-1
shZEB1-2
shCON
shZEB1-1
shZEB1-2
ZEB1
1.00 0.36 0.41
1.00 0.28 0.34
200kDa
HMOX1
1.00 0.36 0.25
1.00 0.30 0.29
30kDa
β-actin
42kDa
CFPAC-1
Capan-1
CFPAC-1
250kDa
150kDa
100kDa
ZEB1
Capan-1
250kDa
150kDa
100kDa
ZEB1
CFPAC-1
35kDa
25kDa
HMOX1
Capan-1
35kDa
25kDa
HMOX1
CFPAC-1
70kDa
50kDa
40kDa
35kDa
β-actin
Capan-1
70kDa
50kDa
40kDa
35kDa
β-actin

Figure 5

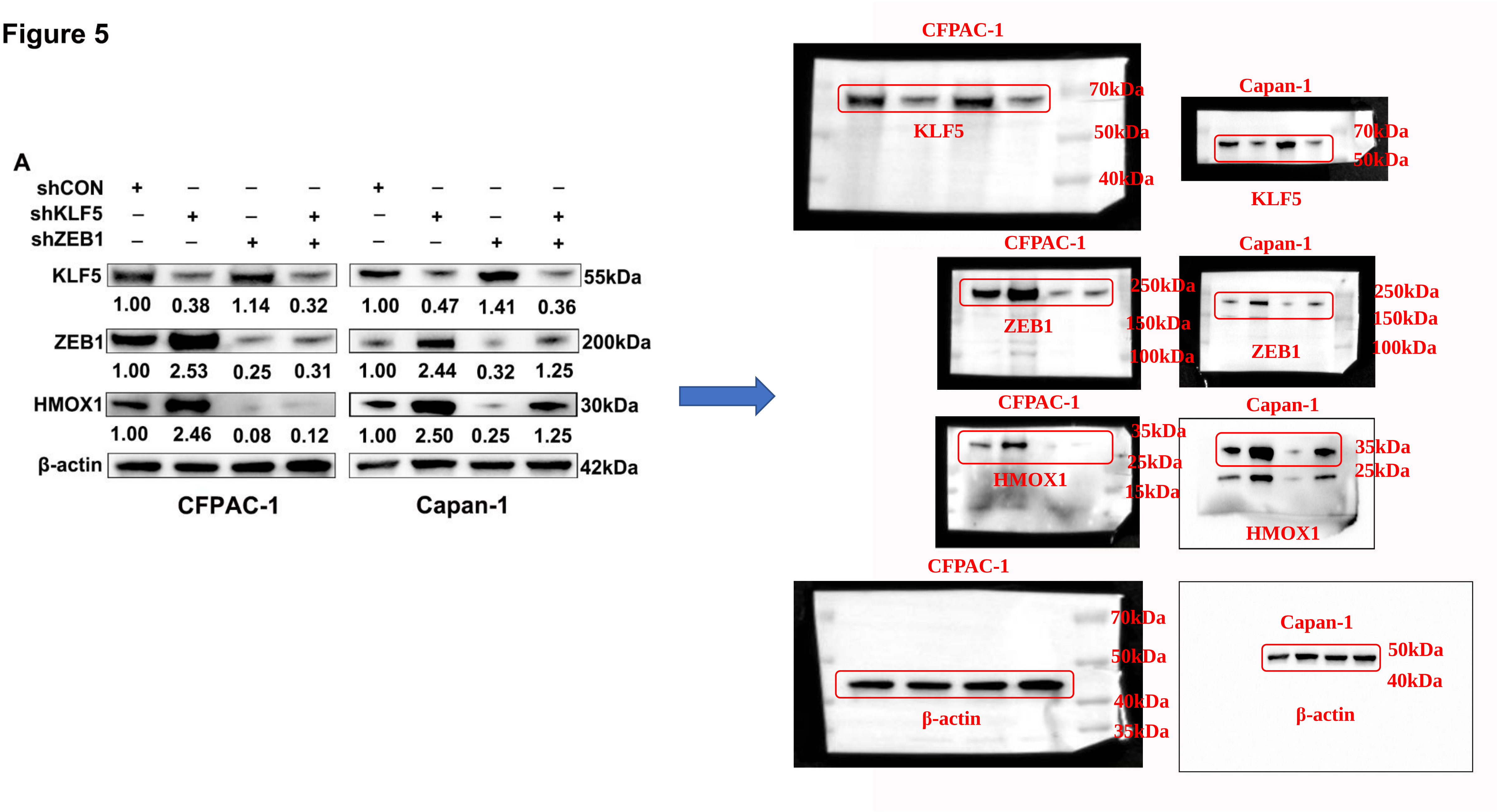

A
shCON + − − − + − − −
shKLF5 − + − + − + − +
shZEB1 − − + + − − + +
KLF5
1.00 0.38 1.14 0.32 1.00 0.47 1.41 0.36
55kDa
ZEB1
1.00 2.53 0.25 0.31 1.00 2.44 0.32 1.25
200kDa
HMOX1
1.00 2.46 0.08 0.12 1.00 2.50 0.25 1.25
30kDa
β-actin
42kDa
CFPAC-1
Capan-1
CFPAC-1
70kDa
50kDa
40kDa
KLF5
Capan-1
70kDa
50kDa
KLF5
CFPAC-1
250kDa
150kDa
100kDa
ZEB1
Capan-1
250kDa
150kDa
100kDa
ZEB1
CFPAC-1
35kDa
25kDa
15kDa
HMOX1
Capan-1
35kDa
25kDa
HMOX1
CFPAC-1
70kDa
50kDa
40kDa
35kDa
β-actin
Capan-1
50kDa
40kDa
β-actin

Figure S2

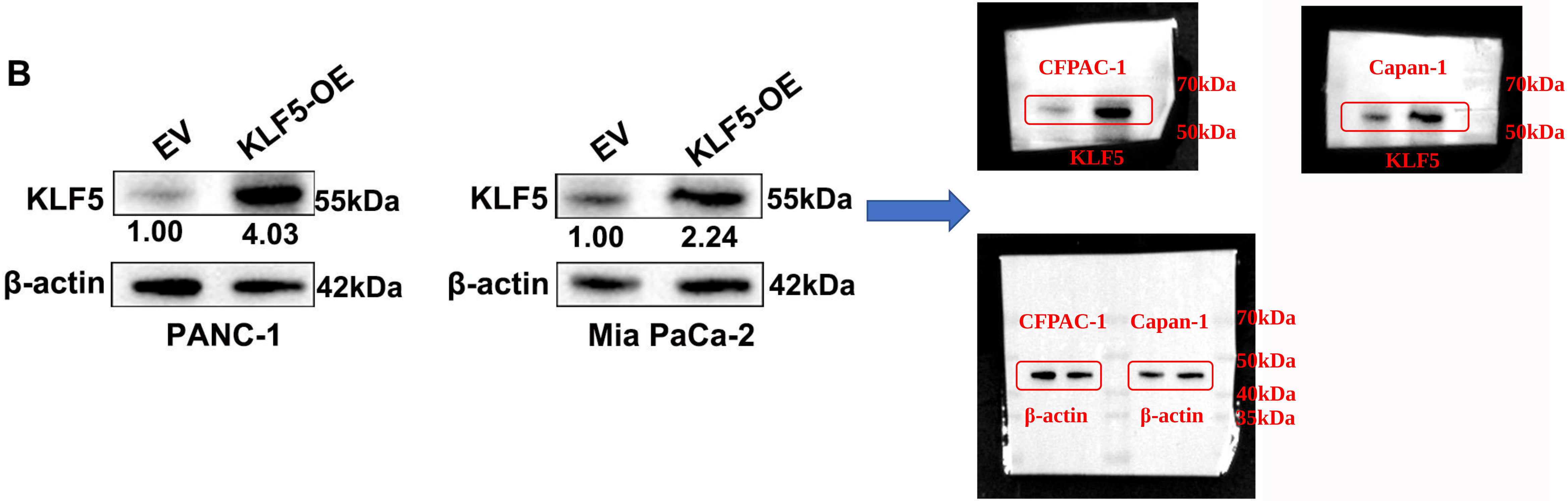
B
EV
KLF5-OE
KLF5
55kDa
1.00
4.03
β-actin
42kDa
PANC-1
EV
KLF5-OE
KLF5
55kDa
1.00
2.24
β-actin
42kDa
Mia PaCa-2
CFPAC-1
70kDa
50kDa
KLF5
Capan-1
70kDa
50kDa
KLF5
CFPAC-1
Capan-1
70kDa
50kDa
40kDa
35kDa
β-actin
β-actin

Figure S3

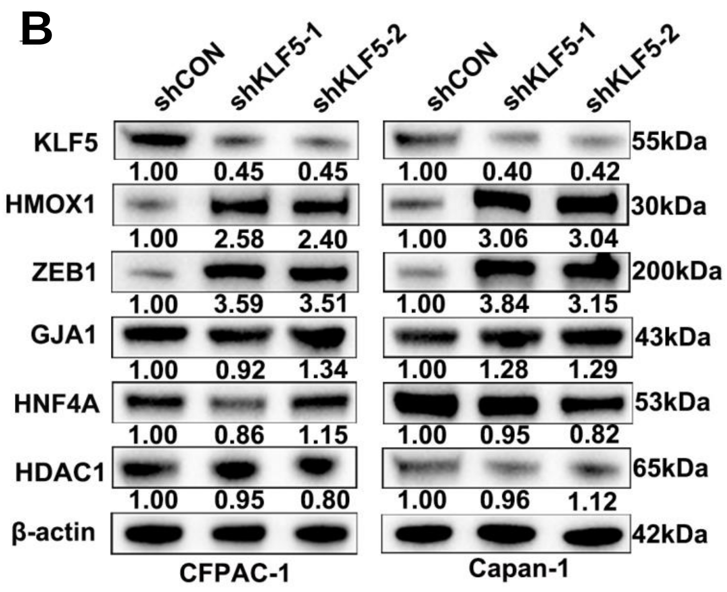
B
shCON
shKLF5-1
shKLF5-2
shCON
shKLF5-1
shKLF5-2
KLF5
1.00 0.45 0.45
1.00 0.40 0.42
55kDa
HMOX1
1.00 2.58 2.40
1.00 3.06 3.04
30kDa
ZEB1
1.00 3.59 3.51
1.00 3.84 3.15
200kDa
GJA1
1.00 0.92 1.34
1.00 1.28 1.29
43kDa
HNF4A
1.00 0.86 1.15
1.00 0.95 0.82
53kDa
HDAC1
1.00 0.95 0.80
1.00 0.96 1.12
65kDa
β-actin
42kDa
CFPAC-1
Capan-1

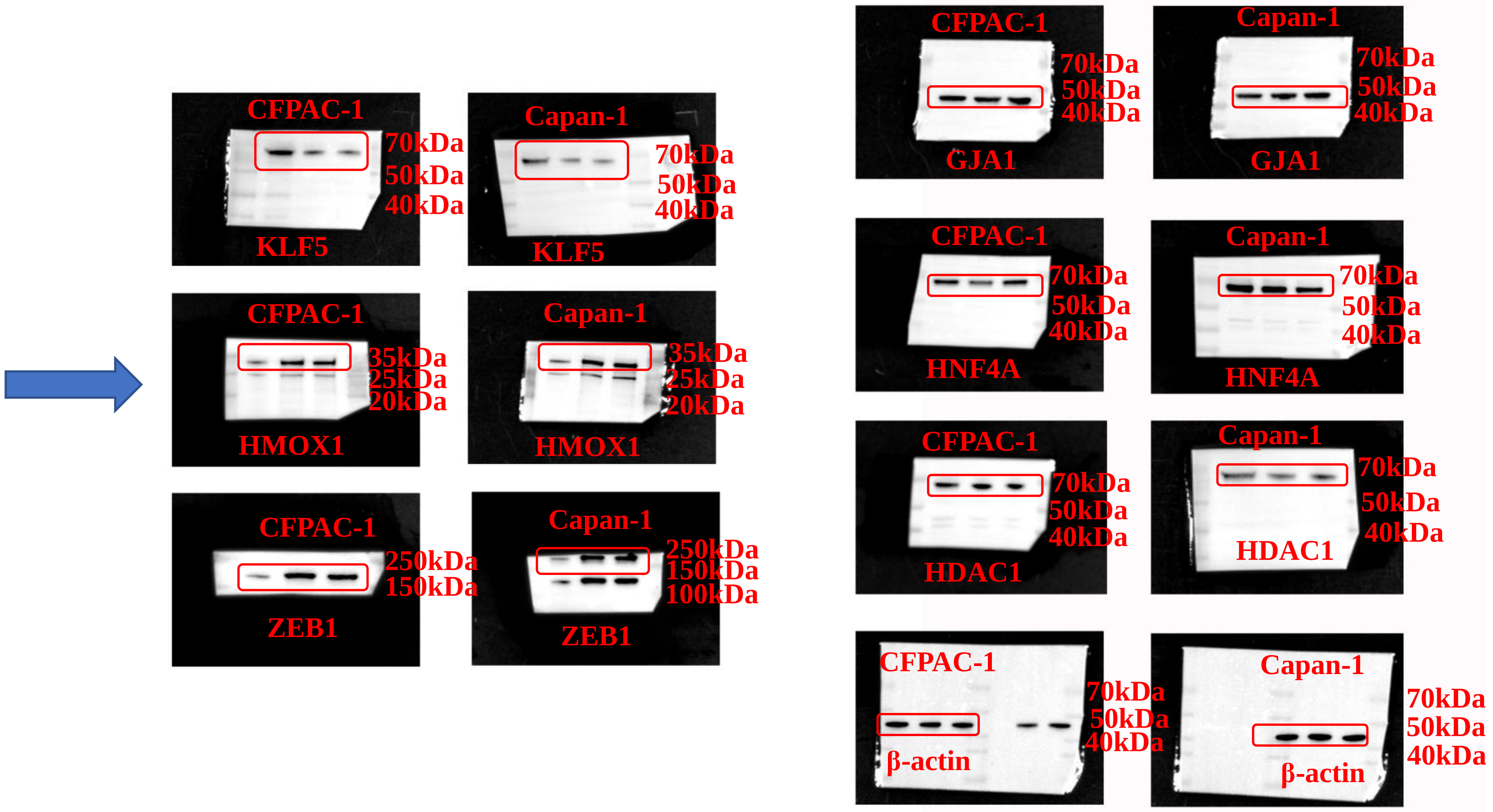
CFPAC-1
KLF5
70kDa
50kDa
40kDa
Capan-1
KLF5
70kDa
50kDa
40kDa
CFPAC-1
HMOX1
35kDa
25kDa
20kDa
Capan-1
HMOX1
35kDa
25kDa
20kDa
CFPAC-1
ZEB1
250kDa
150kDa
Capan-1
ZEB1
250kDa
150kDa
100kDa
CFPAC-1
GJA1
70kDa
50kDa
40kDa
Capan-1
GJA1
70kDa
50kDa
40kDa
CFPAC-1
HNF4A
70kDa
50kDa
40kDa
Capan-1
HNF4A
70kDa
50kDa
40kDa
CFPAC-1
HDAC1
70kDa
50kDa
40kDa
Capan-1
HDAC1
70kDa
50kDa
40kDa
CFPAC-1
β-actin
70kDa
50kDa
40kDa
Capan-1
β-actin
70kDa
50kDa
40kDa

Figure S6

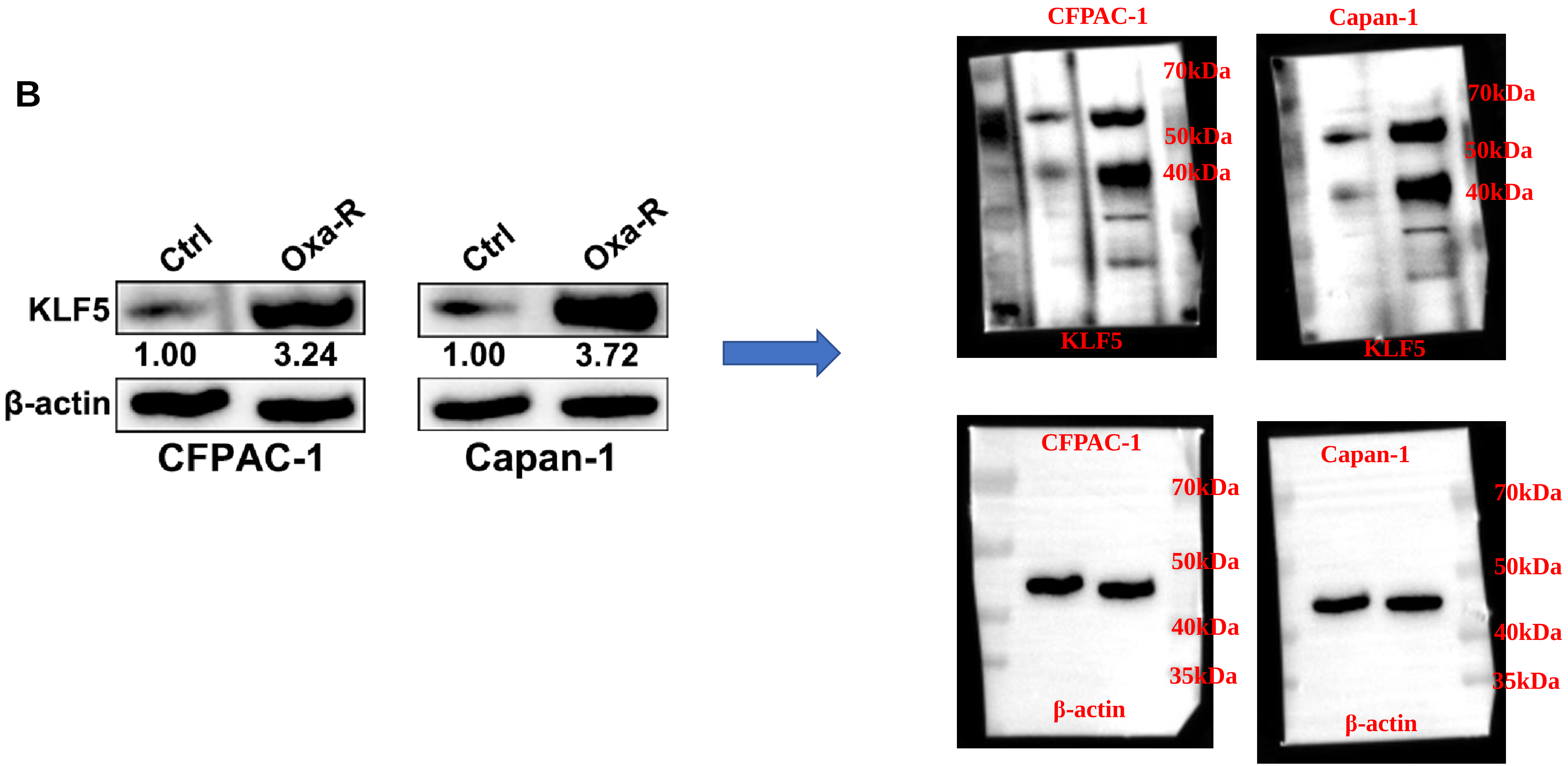
B
Ctrl
Oxa-R
Ctrl
Oxa-R
KLF5
1.00
3.24
1.00
3.72
β-actin
CFPAC-1
Capan-1
CFPAC-1
Capan-1
70kDa
50kDa
40kDa
KLF5
70kDa
50kDa
40kDa
KLF5
CFPAC-1
70kDa
50kDa
40kDa
35kDa
β-actin
Capan-1
70kDa
50kDa
40kDa
35kDa
β-actin
